# IgG and IgM Seroreactivity Against Natural HPV16 Infection and *HLA‐DRB1* and *‐DQB1* Polymorphism

**DOI:** 10.1002/jmv.70471

**Published:** 2025-07-02

**Authors:** Letícia Boslooper Gonçalves, Andrea Trevisan, Eduardo L. Franco, Luisa L. Villa, Helen Trottier, Patrícia Savio de Araujo‐Souza, For the Ludwig‐McGill Study Group

**Affiliations:** ^1^ Post‐Graduation Program in Genetics, Department of Genetics Universidade Federal do Paraná (UFPR) Curitiba Brazil; ^2^ Laboratory of Immunogenetics and Histocompatibility, Department of Genetics Universidade Federal do Paraná (UFPR) Curitiba Brazil; ^3^ Sainte‐Justine Hospital Research Center Université de Montréal Montreal Canada; ^4^ Department of Social and Preventive Medicine, School of Public Health Université de Montréal Montreal Canada; ^5^ Division of Cancer Epidemiology McGill University Montreal Canada; ^6^ Instituto do Cancer do Estado de São Paulo Universidade de São Paulo Sao Paulo Brazil; ^7^ Department of Radiology and Oncology, Faculdade de Medicina Universidade de São Paulo Sao Paulo Brazil

**Keywords:** antibody, HLA polymorphism, HPV16, IgG, IgM

## Abstract

Little is known about the factors associated with the natural humoral immune response against human papillomavirus (HPV) infection. The association between *HLA‐DRB1* and *‐DQB1* polymorphism and (1) HPV16 DNA detection, (2) naturally developed humoral immune response (IgG and IgM) against HPV16 L1 and L2 capsid proteins, and (3) type of HPV16 infection (transient or persistent) was investigated. Data from 943 women participating in the Ludwig‐McGill cohort study was analyzed. *HLA‐DRB1* and *HLA‐DQB1* genotyping was done by PCR‐based methods. HPV DNA was assessed every 4 months during the first year of follow‐up, and HPV16 IgG and IgM antibody measurements were performed by ELISA in serum samples from the first and/or second visits. Associations were estimated by adjusted logistic regression models. HLA‐DRB1*03:02 was associated with HPV16 DNA positivity (OR = 2.49, 95% CI: 1.11–5.60). HLA‐DRB1*13:01 and HLA‐DQB1*04:02 were associated with low levels of HPV16 IgG antibodies (OR = 0.44, 95% CI:0.23–0.87; OR = 0.10, 95% CI: 0.02–0.55, respectively). Neither IgM HPV16 antibodies nor the type of HPV16 infection (transient or persistent) were associated with *HLA‐DRB1* or *‐DQB1* variants. *HLA‐DRB1* and *‐DQB1* polymorphism might be associated with different levels of IgG against HPV16 infection, but IgM production and the type of infection seem to occur independently of these variants.

## 1 Introduction

Human papillomavirus (HPV) ranks as the most common sexually transmitted virus worldwide. More than 90% and 80% of sexually active men and women, respectively, will be infected by HPV in their lifetime [1]. About 90% of HPV infections are transient and clear within 12–24 months [2], but infections from high oncogenic risk HPV (HR‐HPV) genotypes that persist can raise the risk of cervical precancerous lesions, which if left untreated can progress to cancer [3]. More than 200 HPV genotypes have been identified and characterized [4]. However, HPV16 is the main oncogenic genotype, responsible for over 50% of cervical cancers worldwide [3], and is also the most common type found in normal cervix [5].

During primary HPV infection, the cellular immune response is activated mainly via local antigen‐presenting cells (APCs) of the infected epithelium [6]. These APCs present antigens via HLA class I and II molecules to CD8 and CD4 T lymphocytes, respectively. The CD4 T lymphocytes will promote the activation of CD8 T cytotoxic lymphocytes and B cell‐derived humoral immune response. The interaction between naïve B cells and CD4 T lymphocytes leads to B cell activation and antibody production [7]. IgM is the first isotype produced during primary infections [8], followed by IgG, the isotype with the highest plasma concentration [7]. However, naturally developed HPV16 IgG antibodies seem to provide only moderate protection against subsequent HPV16 DNA detection in young women (RR (95% CI) = 0.65 (0.55–0.74)), and not in women aged over 25 years (RR (95% CI) = 0.88 (0.73 –1.04) [9]. IgG seroreactivity to HPV16 has even been considered a marker of past or latent HPV16 infections among older women [10].

HLA classical class II molecules are coded by *HLA‐DRA1, HLA‐DRB1, HLA‐DQA1, HLA‐DQB1, HLA‐DPA1*, and *HLA‐DPB1* genes, located on the short arm of chromosome 6 (6p21.3) [11]. Due to its high genetic variability, essential role in antigen presentation, and consequent involvement, in mediating the immune response, numerous studies have investigated the association between *HLA* class II loci and cervical cancer, mainly exploring *HLA‐DRB1* and *HLA‐DQB1* genes. The HLA‐DRB1*04, HLA‐DRB1*07, HLA‐DRB1*11, HLA‐DRB1*15, HLA‐DQB1*03, and HLA‐DQB1*06:02 were previously shown to be associated with susceptibility to squamous cervical cancer (SCC) [12]. In contrast, HLA‐DRB1*13:01 has been associated with lower risk of cervical cancer [13].

The possible influence of *HLA* polymorphism on the humoral immune response to HPV16 infection remains poorly understood. Our objective was to investigate whether *HLA* class II alleles influence HPV16 infection outcome. More specifically, we analyzed the associations between *HLA‐DRB1* and *‐DQB1* alleles and (1) HPV16 DNA detection, (2) naturally developed HPV16 IgG and IgM antibodies, and (3) type of HPV16 infections (transient or persistent).

## 2 Materials and Methods

### 2.1 Study Design

The Ludwig‐McGill cohort study is a prospective investigation of the natural history of HPV infection [14]. In brief, the cohort included 2462 women recruited from 1993 to 1997—before the implementation of HPV vaccination programs ‐ and followed up for 10 years. The women were recruited from a maternal and child health program for low‐income families at a public hospital in Sao Paulo, Brazil. To be included in this study, women had to be permanent residents in Sao Paulo, aged between 18 and 60 years old; had an intact uterus without previous reported treatment of cervical abnormalities in the past 6 months; no current indication for hysterectomy; no current pregnancy and no intention to become pregnant during the first year of follow‐up; and have reported no use of vaginal medication up to 2 days before data collection. In the first year, blood and cervical cell samples were collected every 4 months for serology testing and cytology and HPV DNA testing, respectively. During the following years, cervical specimens were collected 6 months apart, and HPV16 serology was tested annually. Sociodemographic and behavioral information—such as sexual and contraceptive characteristics ‐ were obtained by questionnaires at every visit. This study was approved by the institutional ethical and research review boards of the participating institutions in Canada and Brazil.

### 2.2 HPV Genotyping

DNA from ectocervical and endocervical cells was extracted and purified using previously described standard techniques [14]. Afterwards, HPV DNA positivity was tested using a PCR protocol which amplifies a L1 viral gene conserved fragment of 450 bp flanked by MY09/11 and PGMY09/11 primers [15, 16]. HPV genotyping was obtained by hybridization with individual oligonucleotide probes for high or intermediate oncogenic risk genotypes (16, 18, 31, 33, 35, 39, 45, 51, 52, 56, 58, 59, 66, 68, 73, and 82) and low oncogenic risk genotypes (6, 11, 26, 32, 34, 40, 42, 44, 53, 54, 57, 61, 62, 64, 67, 69, 70, 71, 72, 81, 83, 84, and 89 [17]). In case of ambiguity, the results were confirmed by restriction fragment length polymorphism analysis of the L1‐amplified fragment using a set of restriction enzymes [14].

### 2.3 HPV Serology

Blood samples were centrifuged to separate the serum, where anti‐HPV16 IgM and IgG antibodies were quantified by ELISA protocol based on genotype‐specific L1 + L2 virus‐like particles (VLPs). Recombinant HPV16 virus‐like particles (VLPs) expressing L1 and L2 (in a baculovirus‐expression system) were kindly provided by Dr. John Schiller, National Institute of Health, United States. Normalized absorbance ratio (NAR) ‐ a standardized procedure used to reduce measurement errors in ELISAs ‐ was used to express seroreactivity, in which the mean blank‐subtracted optical densities (ODs) was divided by the equivalent value of the control serum pool included in the same plate in triplicate, using 1:10 and 1:50 dilutions [18]. HPV16 IgG seroreactivity was measured from samples collected in the first two visits (at 0 and 4 months), and IgM was measured at the first visit (baseline).

### 2.4 *HLA* Genotyping

*HLA* class II genotyping was performed by previously reported PCR‐based methods [19, 20]. Briefly, the second exon of *HLA‐DRB1* and *‐DQB1 loci* were amplified and visualized by electrophoresis 1.5% agarose gel colored with ethidium bromide. Dot‐blotted PCR products were hybridized with sequence‐specific 3′ biotin‐labeled oligonucleotide probes to differentiate *HLA* alleles. The hybridized probes were then detected by chemiluminescence with the ECL kit (Amersham Pharmacia Biotech) after incubation with streptavidin‐peroxidase conjugate. The complete genotypic data for all individuals genotyped at the *HLA‐DRB1* and *‐DQB1 loci* are available in Supporting Information S1, irrespective of inclusion in the present study.

### 2.5 Statistical Analyses

The analyses were restricted to HPV16 seroreactivity due to its relevance in cervical lesions and carcinogenesis. They were based on a subcohort of women tested for both *HLA‐DRB1* and *‐DQB1* (*n* = 948) and also for HPV16 IgG serology (*n* = 943), which was enriched to include women with HPV outcomes as previously described [10, 20]. From the group of women tested for anti‐HPV16 IgG antibodies, 476 were also tested for anti‐HPV16 IgM antibodies.

The associations between *HLA‐DRB1* and *HLA‐DQB1* alleles and (1) HPV16 positivity, (2) anti‐HPV16 IgG and IgM seroreactivity, and (3) type of HPV16 infection (transient or persistent) were evaluated using logistic regression models, which estimated odds ratios (OR) and their respective 95% confidence intervals (CI). Only *HLA‐DRB1* alleles with a frequency ≥ 5% and *HLA‐DQB1* with a frequency ≥ 10% were analyzed. The association between *HLA‐DRB1* and *‐DQB1* and HPV16 DNA positivity was restricted to those women who completed the first year of the study. The estimations were adjusted for race (white vs. non‐white) and age (continuous).

The association between *HLA* alleles and HPV16 IgG and IgM seroreactivity was evaluated using three models. The comparisons were based on NAR values: above versus below median (model 1) and upper tertile versus lower tertile (model 2). These models were restricted to women with the highest probability of having an HR‐HPV infection. The same cutoff of model 2 was used in model 3, restricting the analysis to women who tested positive for HPV16 during the first year of follow‐up, and the ORs were adjusted for race (white vs. non‐white) and age (continuous). For IgG, the highest NAR value obtained between serum dilutions 1:10 and 1:50 tested on the first and second visits were considered. Only the highest NAR value obtained after testing both serum dilutions (1:10 and 1:50) on the first visit was considered for IgM.

To select women who had the highest probability of having an HR‐HPV infection, a propensity score based on the probability of detecting HPV DNA during the first year was estimated, considering the following variables measured at baseline: age (continuous), race (categorical: white vs. non‐white, as inferred by skin color), marital status (categorical: single, married, widowed, separated, cohabiting), age at the first intercourse (continuous), lifetime sexual partners (continuous), and condom use (categorical: always, sometimes, never). Women above the median of the propensity score were considered to have a high risk of HPV infection.

Finally, the association between *HLA‐DRB1* and *‐DQB1* alleles and the type of HPV16 infection (transient or persistent) was evaluated using multinominal logistic regression. In this analysis, women with persistent infection (defined as at least two positive results for HPV16 during the first year of follow‐up) and women with transient infections (defined as one positive result for HPV16 during the first year) were compared to the referent group defined as women who were consistently negative for HPV16 during the first year of follow‐up. The analyses were performed using STATA statistical software version 17 [21].

## 3 Results

Sociodemographic and behavioral characteristics from the entire Ludwig‐McGill cohort (*n* = 2462) and subsets of women tested for both *HLA‐DRB1* and *‐DQB1* and for IgG seroreactivity (*n* = 943) or IgM seroreactivity data (*n* = 476) are described in Table 1. Overall, the distribution of characteristics was similar among groups, except that high‐risk HPV genotypes and the presence of lesions (HSIL) were slightly more common in the subsets.

**Table 1:** Characteristics of the Ludwig‐McGill cohort participants at baseline.

Characteristics at baseline	All participants (*n* = 2462)	Subset tested for *HLA* genotyping (‐*DRB1* and ‐*DQB1*) (*n* = 948)	Subset tested for *HLA* genotyping (‐*DRB1* and ‐*DQB1*) and HPV16 seroreactivity	
			IgG (*n* = 943)	IgM (*n* = 476)
Age, year				
*n*, mean (SD)	2461, 32.7 (8.8)	948, 30.9 (7.7)	943, 30.9 (7.7)	476, 31.0 (8.5)
*n*, median (IQR)	2461, 32.0 (26.0–39.0)	948, 30.0 (25.0–36.0)	943, 30.0 (25.0–36.0)	476, 30.0 (24.0–36.0)
Race, *n* (%)				
White	1585 (64.4)	620 (65.4)	618 (65.5)	313 (65.8)
Non‐white	874 (35.5)	328 (34.6)	325 (34.5)	163 (34.2)
Marital status, *n* (%)				
Single	252 (10.2)	101 (10.7)	101 (10.7)	71 (14.9)
Married	1179 (47.9)	473 (49.9)	472 (50.1)	224 (47.1)
Widowed	57 (2.3)	19 (2.0)	18 (1.9)	10 (2.1)
Separated	140 (5.7)	52 (5.5)	51 (5.4)	33 (6.9)
Cohabiting	832 (33.8)	303 (32.0)	301 (31.9)	138 (29.0)
Income quartiles, *n* (%)				
First quartile	703 (28.6)	267 (28.2)	264 (28.0)	125 (26.3)
Second quartile	530 (21.5)	168 (17.7)	167 (17.7)	57 (12.0)
Third quartile	613 (24.9)	171 (18.0)	171 (18.1)	69 (14.5)
Fourth quartile	615 (25.0)	342 (36.1)	341 (36.2)	225 (47.3)
Education, *n* (%)				
< Elementary	554 (22.5)	195 (20.6)	195 (20.7)	84 (17.7)
Elementary	1438 (58.4)	562 (59.3)	558 (59.2)	290 (60.9)
High School	397 (16.1)	164 (17.3)	163 (17.3)	86 (18.1)
College/University	70 (2.9)	26 (2.7)	26 (2.8)	15 (3.2)
Ever smoked, *n* (%)				
Never	1168 (47.4)	464 (49.0)	461 (48.9)	228 (47.9)
Current	864 (35.1)	328 (34.6)	327 (34.7)	176 (37.0)
Former	429 (17.4)	156 (16.5)	155 (16.4)	72 (15.1)
Ever drank alcohol occasionally, *n* (%)				
Never	852 (34.6)	305 (32.2)	303 (32.1)	150 (31.5)
Ever drinker	1601 (65.0)	642 (67.7)	639 (67.8)	326 (68.5)
Lifetime *n* ^o^ of sexual partners, *n* (%)				
0–1	1,089 (44.2)	414 (43.7)	412 (43.7)	190 (39.9)
2–3	856 (34.8)	344 (36.3)	342 (36.3)	174 (36.6)
4+	515 (20.9)	189 (19.9)	188 (19.9)	112 (23.5)
Age at first sexual intercourse, year				
*n*, mean (SD)	2,461, 17.9 (4.0)	948, 17.6 (3.6)	943, 17.6 (3.6)	476, 17.5 (3.8)
*n*, median (IQR)	2,461, 17.0 (15.0–20.0)	948, 17.0 (15.0–19.0)	943, 17.0 (15.0–19.0)	476, 17.0 (15.0–19.0)
Parity				
*n*, mean (SD)	2,338, 2.3 (2.1)	888, 2.2 (1.9)	883, 2.2 (1.9)	437, 2.3 (2.0)
*n*, median (IQR)	2,338, 2.0 (1.0–3.0)	888, 2.0 (1.0– 3.0)	883, 2.0 (1.0–3.0)	437, 2.0 (1.0–3.0)
Ever used oral contraceptive, *n* (%)				
Never	401 (16.3)	136 (14.4)	133 (14.1)	69 (14.5)
0–5 years	1,349 (54.8)	556 (58.7)	555 (58.9)	278 (58.4)
≥ 6 years	711 (28.9)	256 (27.0)	255 (27.0)	129 (27.1)
Ever had STD, *n* (%)				
Never	1,881 (76.4)	732 (77.2)	729 (77.3)	361 (75.8)
Condyloma or venereal warts	108 (4.4)	53 (5.6)	53 (5.6)	36 (7.6)
Others^a^	463 (18.8)	160 (16.9)	158 (16.8)	78 (16.4)
HPV DNA status, *n* (%)				
HPV negative	2,026 (82.3)	745 (78.6)	741 (78.6)	330 (69.3)
Positive for low‐risk genotypes	156 (6.3)	76 (8.0)	75 (8.0)	60 (12.6)
Positive for HPV16	67 (2.7)	36 (3.8)	36 (3.8)	30 (6.3)
Positive for HPV31 or 35	37 (1.5)	19 (2.0)	19 (2.0)	13 (2.7)
Positive for HPV52 or 67 or 33 or 58	46 (1.9)	26 (2.7)	26 (2.8)	18 (3.8)
Positive for other high‐risk genotypes	107 (4.4)	46 (4.9)	46 (4.9)	25 (5.3)
Type of HPV16 infection, *n* (%)				
Single	45 (1.8)	24 (2.5)	24 (2.6)	21 (4.4)
Multiple	22 (0.9)	12 (1.3)	12 (1.3)	9 (1.9)
Number of HPV genotypes detected with HPV16, *n* (%)				
2	17 (77.3)	8 (66.7)	8 (66.7)	7 (77.8)
3+	5 (22.7)	4 (33.3)	4 (33.3)	2 (22.2)
Number of HPV genotypes detected, *n* (%)				
0	2,026 (82.3)	745 (78.6)	741 (78.6)	330 (69.3)
1	336 (13.7)	163 (17.2)	162 (17.2)	123 (25.8)
2	63 (2.6)	32 (3.4)	32 (3.4)	19 (4.0)
3+	14 (0.6)	8 (0.9)	8 (0.9)	4 (0.8)
Cytology, *n* (%)				
Negative	2,361 (95.9)	883 (93.1)	880 (93.3)	434 (91.2)
ASCUS/AGUS	43 (1.8)	22 (2.3)	21 (2.2)	15 (3.2)
LSIL	31 (1.3)	21 (2.2)	21 (2.2)	9 (1.9)
HSIL	21 (0.9)	21 (2.2)	20 (2.2)	18 (3.8)
HPV16 seroreactivity, NAR				
*n*, mean (SD)	NA	NA	943, 0.9 (0.6)	476, 1.2 (0.4)
*n*, median (IQR)	NA	NA	943, 0.8 (0.5–1.2)	476, 1.1 (0.9–1.3)

Abbreviations: AGUS, atypical glandular cells of undermined significance; ASCUS, atypical squamous cells of undermined significance; CPC, viral copies per cell; HSIL, high grade squamous intraepithelial lesion; IQR, interquartile range; LSIL, low grade squamous intraepithelial lesion; NA, not applicable; NAR, normalized absorbance ratio; SD, standard deviation; STD, sexually transmitted disease; yr, years.

^a^ Gonorrhea, chancre, syphilis, herpes, trichomoniasis, candidiasis.

Table 2 shows the associations between *HLA* alleles and HPV16 positivity, IgG, and IgM seroreactivity. An association between HLA‐DRB1*03:02 and HPV16 positivity (OR = 2.49, 95% CI = 1.11–5.60) was observed. HLA‐DRB1*13:01 and HLA‐DQB1*04:02 were associated with low levels of HPV16 IgG seroreactivity (OR = 0.44, 95% CI = 0.23–0.87; OR = 0.10, 95% CI = 0.02–0.55, respectively). No significant associations were observed between IgM seroreactivity and *HLA‐DRB1* and *‐DQB1* alleles.

**Table 2:** Logistic regression analyses with HPV16 positivity, IgG, and IgM HPV16 seroreactivity as the outcomes.

Allele	HPV16 positivity^a^ (*n* = 948)		Model 1^b^				Model 2^c^				Model 3^d^			
			IgG (*n* = 452)		IgM (*n* = 242)		IgG (*n* = 300)		IgM (*n* = 161)		IgG (*n* = 49)		IgM (*n* = 34)	
	OR	95% CI	OR	95% CI	OR	95% CI	OR	95% CI	OR	95% CI	OR	95% CI	OR	95% CI
***HLA‐DRB1***														
DRB1*01:01	0.89	0.35–2.29	1.50	0.74–3.06	1.27	0.44–3.68	1.42	0.61–3.32	1.71	0.41–7.08	NE	NE	NE	NE
DRB1*01:02	0.92	0.36–2.36	0.90	0.47–1.73	0.82	0.31–2.15	0.76	0.34–1.69	0.99	0.29–3.40	NE	NE	2.20	0.17–28.15
DRB1*03:01	0.73	0.34–1.57	1.36	0.82–2.25	1.80	0.83–3.90	1.25	0.66–2.37	1.95	0.79–4.81	1.07	0.10‐11.33	1.46	0.09–24.98
DRB1*03:02	**2.49**	**1.11–5.60**	0.85	0.39–1.88	1.05	0.40–2.75	1.45	0.52–4.03	1.49	0.42–5.31	0.26	0.04‐1.55	NE	NE
DRB1*07:01	0.90	0.50–1.65	0.78	0.51–1.19	1.37	0.75–2.49	0.74	0.44–1.26	1.19	0.57–2.48	0.62	0.12‐3.27	0.62	0.13–3.06
DRB1*11:01	1.34	0.68–2.66	0.88	0.50–1.53	0.57	0.27–1.19	1.11	0.57–2.17	0.46	0.19–1.08	1.96	0.20‐19.13	0.66	0.11–3.91
DRB1*13:01	0.59	0.23–1.51	0.61	0.35–1.07	1.63	0.77–3.47	**0.44**	**0.23–0.87**	1.76	0.63–4.96	0.89	0.08‐10.11	0.46	0.04–6.01
DRB1*13:02	1.19	0.50–2.87	1.81	0.93–3.55	0.90	0.38–2.13	1.92	0.85–4.35	0.45	0.13–1.60	NE	NE	1.04	0.06–19.91
DRB1*15:01	1.18	0.54–2.57	1.23	0.67–2.26	0.87	0.41–1.86	1.62	0.73–3.60	0.80	0.34–1.91	0.28	0.03‐2.27	0.41	0.08–2.16
DRB1*15:03	1.10	0.45–2.69	1.55	0.80–3.01	0.89	0.39–2.04	1.04	0.51–2.12	1.60	0.56–4.55	NE	NE	0.87	0.11–7.10
***HLA‐DQB1***														
DQB1*02:01	0.71	0.41–1.22	0.87	0.60–1.27	1.52	0.89–2.57	0.86	0.54–1.37	1.36	0.72–2.56	0.67	0.15–2.98	0.48	0.09–2.50
DQB1*03:01	1.27	0.74–2.19	0.81	0.54–1.21	0.66	0.38–1.14	1.03	0.61–1.72	0.76	0.39–1.47	1.30	0.25–6.69	1.88	0.44–7.93
DQB1*03:02	0.79	0.35–1.77	0.72	0.42–1.24	1.08	0.51–2.29	0.58	0.29–1.17	0.72	0.28–1.87	NE	NE	0.32	0.02–4.36
DQB1*04:02	1.71	0.92–3.22	0.97	0.57–1.65	1.06	0.55–2.07	0.96	0.51–1.82	1.05	0.46–2.40	**0.10**	**0.02–0.55**	1.45	0.25–8.25
DQB1*05:01	0.88	0.48–1.60	1.24	0.81–1.90	1.56	0.84–2.91	1.12	0.68–1.87	1.66	0.74–3.74	6.35	0.66–61.15	1.02	0.16–6.54
DQB1*06:02	0.81	0.43–1.53	1.32	0.85–2.06	0.88	0.50–1.55	1.22	0.72–2.08	1.16	0.59–2.27	0.74	0.12–4.71	0.36	0.08–1.63
DQB1*06:03	0.74	0.29–1.90	0.69	0.37–1.29	1.13	0.51–2.50	0.57	0.27–1.19	1.12	0.37–3.38	NE	NE	1.87	0.14–24.80

*Note:* Bold values are statistically significant at the alpha level of 0.05.

Abbreviation: NE, not estimated.

^a^ HPV16 positivity during the first year of follow‐up, adjusted for race (white and nonwhite) and age (continuous).

^b^ Model 1: serology categorized using the median (above vs. below), restricted by the propensity score for high risk of HR‐HPV infection.

^c^ Model 2: serology highest tertile versus lowest tertile, restricted by the propensity score for high risk of HR‐HPV infection (equal or above the median).

^d^ Model 3: serology highest tertile versus lowest tertile adjusted for race (white and nonwhite) and age (continuous) and restricted by HPV16 positivity during the first year of follow‐up.

Associations between *HLA‐DRB1* and *‐DQB1* polymorphism, and type of HPV16 infection are presented in Table 3. No significant associations were observed for transient or persistent HPV16 infections and the *HLA* alleles.

**Table 3:** Multinomial logistic regression analysis with transient and persistent HPV16 infection as the outcomes.

Allele	Transient infection^a^ (*n* = 919)		Persistent infection^a^ (*n* = 919)	
	OR	95% CI	OR	95% CI
***HLA‐DRB1***				
DRB1*01:01	0.86	0.20–3.71	0.81	0.19–3.50
DRB1*01:02	1.40	0.41–4.78	0.88	0.20–3.77
DRB1*03:01	0.20	0.03–1.49	1.14	0.43–3.07
DRB1*03:02	3.01	0.98–9.29	2.25	0.64–7.88
DRB1*07:01	1.07	0.44–2.58	0.80	0.32–2.02
DRB1*11:01	1.13	0.38–3.35	2.18	0.90–5.30
DRB1*13:01	0.90	0.27–3.07	0.26	0.04–1.92
DRB1*13:02	1.45	0.42–4.95	0.91	0.21–3.91
DRB1*15:01	1.59	0.53–4.79	1.40	0.47–4.17
DRB1*15:03	1.25	0.35–4.38	0.86	0.19–3.79
***HLA‐DQB1***				
DQB1*02:01	0.52	0.22–1.25	0.96	0.44–2.10
DQB1*03:01	1.07	0.46–2.50	1.60	0.73–3.51
DQB1*03:02	0.55	0.13–2.35	1.45	0.54–3.91
DQB1*04:02	1.44	0.53–3.91	1.80	0.71–4.56
DQB1*05:01	1.02	0.42–2.47	0.64	0.24–1.72
DQB1*06:02	1.11	0.45–2.70	0.72	0.27–1.93
DQB1*06:03	0.73	0.17–3.16	0.67	0.16–2.88

^a^ Adjusted for race (white and non‐white) and age (continuous) and restricted to women tested for *HLA‐DRB1* and *‐DQB1*. Only women with four visits during the first year were considered.

## 4 Discussion

HLA class II molecules may play a role in the antibody immune response against HPV infection, considering that T‐follicular helper (Tfh) cells, a subset of CD4 T cells, help antigen‐primed B lymphocytes differentiate into plasma cells and secrete antibodies [22]. The interaction between the HLA class II molecule–peptide complex and the T‐cell receptor is necessary for CD4 T‐cell activation and to build an effective humoral response. Therefore, it is reasonable to hypothesize that differences in HLA class II molecules caused by genetic polymorphism may affect the efficacy in binding and presenting HPV16 antigens to lymphocytes, affecting the humoral immune response.

Although several studies have investigated the associations between *HLA* alleles and the susceptibility to HPV‐associated diseases [23, 24, 25, 26, 27, 28, 29], the mechanism behind this potential relationship is still unknown. It was previously showed that patients with certain haplotypes are less likely to clear HPV infection [12]. Some studies have also analyzed the relationship between *HLA* polymorphism and the serological immune response to HPV infections. A genome‐wide association study (GWAS) identified a common genetic variant (rs9357152–A/G), located on chromosome 6p21.32 in the major histocompatibility complex (MHC) class II region nearby gene *HLA‐DQB1*, associated with HPV8 L1 seropositivity in Europeans [30]. Moreover, it was further observed in this same cohort that HLA‐DRB1*11:01 and ‐DQB1*03:01 were associated with HPV8 seropositivity, and HLA‐DRB1*07:01 and ‐DQA1*02:01 were associated with HPV77 seropositivity [31]. It is important to highlight that even though all HPVs infect cells in the basal layer of stratified epithelia, HPV8 and HPV77 are cutaneous genotypes and are not involved in cervical infections [32]. Additionally, a recent study showed that antibody levels against HPV16 L1 and E6 viral proteins measured in HPV+ oropharyngeal cancer (HPV( + )OPC) patients were affected by *HLA* variants [33]. The *locus* rs4713462 was associated with antibody levels against HPV16 E6, and an amino acid variation in HLA‐DRβ1 (71‐Glu) was associated with antibody levels against HPV16 L1, the viral capsid protein [33]. Despite these investigations, none of them analyzed the association between *HLA* variants and HPV seroreactivity in the context of cervical lesions.

In our study, associations between HLA‐DRB1*13:01 and HLA‐DQB1*04:02 and low levels of HPV16 IgG seroreactivity were observed, indicating a decreased likelihood of antibody response against HPV16 with these alleles. Contradictorily, HLA‐DRB1*13 was associated with a reduced risk of cervical cancer in different populations pooled in a meta‐analysis [13] while no association was found with HPV infection (transient or persistent infections with any genotypes) in the Ludwig‐McGill cohort [20]. Our findings also showed that neither transient nor persistent HPV16 infections were affected by *HLA‐DRB1* and *‐DQB1* polymorphisms. Similarly, another cohort study showed no evidence of association between HPV persistence and *HLA* alleles, although only a few alleles (HLA‐B*07, DQB1*03, DQB1*06:02, DRB1*13, and DRB1*15:01) or haplotypes were considered [34]. A previous analysis from the Ludwig‐McGill cohort examining the risk of HPV persistence for all genotypes combined (not limited to HPV16) showed that HLA‐DRB1*03:01‐DQB1*02:01 haplotype was associated with lower risk of transient and persistent HPV infections, HLA‐DRB1*11:02‐DQB1*03:01 with a lower risk of HPV persistence and HLA‐DRB1*16:01‐DQB1*05:02 and HLA‐DRB1*08:07‐DQB1*04:02 with a higher risk of HPV persistence [19].

It should be recognized that the associations found in studies investigating *HLA* may be more likely to be observed by chance given the large number of statistical tests that are typically performed. However, it is important to note that our analysis was performed based on *HLA* alleles, not haplotypes, only considering HPV16, and including a large sample size. Thus, the results of our study apply to HPV16 and may not generalize to all HPV genotypes. Furthermore, our study included women with a mean age of 33 years. Expression of HLA molecules in the cell surface, as well as the serology levels may be different in these women compared to younger women. Indeed, studies have shown that natural antibodies developed against HPV16 have a moderate protective effect against subsequent HPV detection in younger women (under 25‐30 years), while it has been associated with an increased risk of HPV outcomes in older women [9, 10]. It is therefore possible that the effect of HLA observed in our study generalizes only to older women.

This study has several strengths. IgG and IgM antibodies against HPV were measured from a large sample of unvaccinated women, which provides insight into the development of natural infections. Also, testing was done using the most common method to test HPV serology in the literature, with all assays performed using the same technology at the same laboratory by two trained students. However, even though the study had a meticulous data collection protocol, the participants’ history of past HPV infection was unknown. It is impossible to determine if the antibody levels detected at that time point were from current/recent or past HPV infections. Another limitation of the study is that HPV16 IgG and IgM antibodies were not measured in all follow‐up visits, making it impossible to account for antibody dynamics over time. Finally, although there was an adjustment for potential confounders such as race and age, residual confounding is possible.

In conclusion, these results suggest that *HLA‐DRB1* and *‐DQB1* polymorphism might be associated with different levels of IgG against HPV16 infection, but IgM production and the type of infection (transient or persistent) seem to occur independently of these variants. Further evaluations are required to elucidate the genetic background behind the differences in naturally developed antibody levels against HPV infections.

## Author Contributions

Letícia Boslooper Gonçalves performed the statistical analysis, analyzed the data, and drafted the manuscript. Andrea Trevisan helped with the statistical analysis and revised the manuscript. Helen Trottier supervised the study, helped analyze the data, and critically revised the manuscript. Luisa L. Villa and Eduardo L. Franco contributed to the conceptualization, methodology, project administration, funding acquisition, and manuscript revision. Patrícia Savio de Araujo‐Souza conceived and supervised the study, analyzed the data, and critically revised the manuscript.

## Ethics Statement

Eligible participants signed informed consent form. The study protocol was approved by the ethical review boards of the participating institutions in Canada and Brazil.

## Conflicts of Interest

ELF's institution has received grants from Merck in support of his investigator‐initiated studies. He has also served as an occasional advisor for companies involved with HPV vaccines (Merck, GSK) and HPV diagnostics (Roche, BD). LLV has received institutional grants from Merck to support her investigator‐initiated studies. She is also an occasional advisor and speaker of Merck, Sharp and Dohme for HPV vaccines and HPV diagnostics (Roche, BD. Qiagen, Cepheid). HT has received occasional lecture fees from Merck. The other authors reported no conflict of interest.

## Supporting information

Supporting material 1.

## Data Availability

All relevant data are included in the article. Data files and Stata codes are available upon request.

## References

[1] H. W. Chesson , E. F. Dunne , S. Hariri , and L. E. Markowitz , “The Estimated Lifetime Probability of Acquiring Human Papillomavirus in the United States,” Sexually Transmitted Diseases 41, no. 11 (2014): 660–664. 25299412 10.1097/OLQ.0000000000000193 PMC6745688

[2] E. L. Franco , L. L. Villa , J. P. Sobrinho , et al., “Epidemiology of Acquisition and Clearance of Cervical Human Papillomavirus Infection in Women From a High‐Risk Area for Cervical Cancer,” Journal of Infectious Diseases 180, no. 5 (1999): 1415–1423. 10515798 10.1086/315086

[3] H. Trottier and E. L. Franco , “The Epidemiology of Genital Human Papillomavirus Infection,” Vaccine 24 (2006): S4–S15. 10.1016/j.vaccine.2005.09.054 16406226

[4] International Human Papillomavirus Reference Center [Internet] . [cited 2025 Jan 26], https://www.hpvcenter.se/human_reference_clones/.

[5] L. Bruni , G. Albero , B. Serrano , et al., “ICO/IARC Information Centre on HPV and Cancer (HPV Information Centre),” Human Papillomavirus and Related Diseases in the World. [Internet] (2023): 1–296, www.hpvcentre.net.

[6] R. B. S. Roden and P. L. Stern , “Opportunities and Challenges for Human Papillomavirus Vaccination in Cancer,” Nature Review Cancer 18 4 (2018): 240–254, 10.1038/nrc.2018.13. 29497146 PMC6454884

[7] R. S. Mboumba Bouassa , H. Péré , M. A. Jenabian , et al., “Natural and Vaccine‐Induced B Cell‐Derived Systemic and Mucosal Humoral Immunity to Human Papillomavirus,” Expert Review of Anti‐Infective Therapy 18, no. 6 (2020): 579–607, 10.1080/14787210.2020.1750950. 32242472

[8] K. Jones , A. F. Savulescu , F. Brombacher , and S. Hadebe , “Immunoglobulin M in Health and Diseases: How Far Have We Come and What Next?,” Frontiers in immunology 11, no. October (2020): 1–15. 33193450 10.3389/fimmu.2020.595535 PMC7662119

[9] A. Trevisan , P. S. De araujo‐Souza , A. Pincivy , J. Niyibizi , E. L. Franco , and H. Trottier , “Systematic Review and Meta‐Analysis of the Association Between Naturally Induced IgG, IgM and Neutralising Antibodies to HPV16 and Newly Detected Cervical HPV16 Infection Outcomes,” Sexually Transmitted Infections 101, no. 3 (2025): 191–199, http://sti.bmj.com/content/101/3/191.abstract. 39542714 10.1136/sextrans-2024-056296

[10] A. Trevisan , J. M. G. Candeias , P. Thomann , L. L. Villa , E. L. Franco , and H. Trottier , “Naturally Developed HPV16 Antibodies and Risk of Newly Detected Cervical HPV Infection Outcomes,” Journal of Medical Virology 96, no. 4 (2024): e29608. 38623750 10.1002/jmv.29608 PMC12172024

[11] Complete Sequence and Gene Map of a Human Major Histocompatibility Complex. The MHC Sequencing Consortium,” Nature 401, no. 28 (1999): 921–923. 10553908 10.1038/44853

[12] P. S. de Araujo Souza , L. Sichero , and P. C. Maciag , “HPV Variants and HLA Polymorphisms: The Role of Variability on the Risk of Cervical Cancer,” Future Oncology 5, no. 3 (2009): 359–370. 19374542 10.2217/fon.09.8

[13] A. B. Kamiza , S. Kamiza , and C. G. Mathew , “HLA‐DRB1 Alleles and Cervical Cancer: A Meta‐Analysis of 36 Case‐Control Studies,” Cancer Epidemiology 67, no. May (2020): 101748, 10.1016/j.canep.2020.101748. 32562888

[14] E. Franco , L. Villa , T. Rohan , A. Ferenczy , M. Petzl‐Erler , and G. Matlashewski , “Design and Methods of the Ludwig‐Mcgill Longitudinal Study of the Natural History of Human Papillomavirus Infection and Cervical Neoplasia in Brazil,” Revista Panamericana de Salud Pública 6, no. 4 (1999): 223–233. 10572472 10.1590/s1020-49891999000900001

[15] P. E. Gravitt , C. L. Peyton , T. Q. Alessi , et al., “Improved Amplification of Genital Human Papillomaviruses,” Journal of Clinical Microbiology 38, no. 1 (2000): 357–361. 10618116 10.1128/jcm.38.1.357-361.2000 PMC88724

[16] M. Manos , Y. Ting , W. Dk , L. Aj , and B. Tr , “Use of Polymerase Chain Reaction Amplification for the Detection of Genital Human Papillomaviruses,” Molecular Diagnostics in Human Cancer Cells 7(1989): 209–214.

[17] H. M. Bauer , “Genital Human Papillomavirus Infection in Female University Students as Determined by a PCR‐Based Method,” JAMA: The Journal of the American Medical Association 265, no. 4 (1991): 472–477, 10.1001/jama.1991.03460040048027. 1845912

[18] A. V. Ramanakumar , P. Thomann , J. M. Candeias , S. Ferreira , L. L. Villa , and E. L. Franco , “Use of the Normalized Absorbance Ratio as an Internal Standardization Approach to Minimize Measurement Error in Enzyme‐Linked Immunosorbent Assays for Diagnosis of Human Papillomavirus Infection,” Journal of Clinical Microbiology 48, no. 3 (2010): 791–796. 20053861 10.1128/JCM.00844-09 PMC2832464

[19] P. C. Maciag , N. F. Schlecht , P. S. A. Souza , T. E. Rohan , E. L. Franco , and L. L. Villa , “Polymorphisms of the Human Leukocyte Antigen DRB1 and DQB1 Genes and the Natural History of Human Papillomavirus Infection,” Journal of infectious diseases 186, no. 2 (2002): 164–172. 12134251 10.1086/341080

[20] P. S. De araujo‐Souza , M. El‐Zein , A. N. Bolpetti , et al., “Association Between Human Leukocyte Antigen Polymorphism and Human Papillomavirus Infection in Brazilian Women,” Sexually Transmitted Diseases 50, no. 1 (2023): 50–58. 36194829 10.1097/OLQ.0000000000001718 PMC9742174

[21] StataCorp ., Stata Statistical Software: Release 17 (StataCorp LLC [Internet], 2021), https://www.stata.com/.

[22] C. G. Vinuesa , M. A. Linterman , D. Yu , and I. C. M. Maclennan , “Follicular Helper T Cells,” Annual Review of Immunology 34 (2016): 335–368. 10.1146/annurev-immunol-041015-055605 26907215

[23] S. Ades , A. Koushik , E. Duarte‐Franco , et al., “Selected Class I and Class II HLA Alleles and Haplotypes and Risk of High‐Grade Cervical Intraepithelial Neoplasia,” International Journal of Cancer 122, no. 12 (2008): 2820–2826. 18351579 10.1002/ijc.23459

[24] D. Chen , I. Juko‐pecirep , J. Hammer , et al., “Genome‐Wide Association Study of Susceptibility Loci for Cervical Cancer,” JNCI: Journal of the National Cancer Institute 105, no. 9 (2013): 624–633. 23482656 10.1093/jnci/djt051

[25] L. B. Gonçalves , P. P. de França , N. A. Petry , et al., “Inside the Pocket: Critical Elements of HLA ‐Mediated Susceptibility to Cervical Precancerous Lesions,” HLA 98 (2021): 448–458. 34505756 10.1111/tan.14429

[26] P. Lin , L. A. Koutsky , C. W. Critchlow , et al., “HLA Class II DR‐DQ and Increased Risk of Cervical Cancer Among Senegalese Women,” Cancer Epidemiology, Biomarkers & Prevention: A Publication of the American Association for Cancer Research, cosponsored by the American Society of Preventive Oncology 10, no. 10 (2001): 1037–1045. 11588129

[27] P. C. Maciag , N. F. Schlecht , P. S. Souza , E. L. Franco , L. L. Villa , and M. L. Petzl‐Erler , “Major Histocompatibility Complex Class II Polymorphisms and Risk of Cervical Cancer and Human Papillomavirus Infection in Brazilian Women,” Cancer Epidemiology, Biomarkers & Prevention: A Publication of the American Association for Cancer Research, cosponsored by the American Society of Preventive Oncology 9 (2000): 1183–1191. 11097225

[28] K. Matsumoto , H. Maeda , A. Oki , et al., “Human Leukocyte Antigen Class II DRB1*1302 Allele Protects Against Cervical Cancer: At Which Step of Multistage Carcinogenesis?,” Cancer Science 106, no. 10 (2015): 1448–1454. 26235935 10.1111/cas.12760 PMC4638013

[29] M. Safaeian , L. G. Johnson , K. Yu , et al., “Human Leukocyte Antigen Class I and II Alleles and Cervical Adenocarcinoma,” Frontiers in Oncology 4, no. June (2014): 1–8. 24995157 10.3389/fonc.2014.00119 PMC4062965

[30] D. Chen , J. D. McKay , G. Clifford , et al., “Genome‐Wide Association Study of HPV Seropositivity,” Human Molecular Genetics 20, no. 23 (2011): 4714–4723. 21896673 10.1093/hmg/ddr383

[31] D. Chen , V. Gaborieau , Y. Zhao , et al., “A Systematic Investigation of the Contribution of Genetic Variation Within the MHC Region to HPV Seropositivity,” Human Molecular Genetics 24, no. 9 (2015): 2681–2688. 25616963 10.1093/hmg/ddv015

[32] H. zur Hausen , “Papillomaviruses and Cancer: From Basic Studies to Clinical Application,” Nature Reviews Cancer 2, no. 5 (2002): 342–350. 12044010 10.1038/nrc798

[33] A. Ferreiro‐Iglesias , J. D. McKay , N. Brenner , et al., “Germline Determinants of Humoral Immune Response to HPV‐16 Protect Against Oropharyngeal Cancer,” Nature Communications 12 (2021): 1–11, 10.1038/s41467-021-26151-9. PMC8511029 34642315

[34] S. M. Mahmud , K. Robinson , H. Richardson , et al., “HLA Polymorphisms and Cervical Human Papillomavirus Infection in a Cohort of Montreal University Students,” Journal of Infectious Diseases 196, no. 1 (2007): 82–90. 17538887 10.1086/518612

