# Supplementary material for: IgG and IgM Seroreactivity Against Natural HPV16 Infection and HLA‐DRB1 and ‐DQB1 Polymorphism
Source: J Med Virol. 2025 Jul 2;97(7):e70471. doi: 10.1002/jmv.70471 (PMC12216804; doi:10.1002/jmv.70471)
Supplement: Supplementary file 1 — Supporting material 1. [file JMV-97-e70471-s001.pdf]

Supplementary material 1: *HLA-DRB1* and *-DQB1* genotypes

ID	HLA-DRB1_1	HLA-DRB1_2	HLA-DQB1_1	HLA-DQB1_2
1	DRB1*01:02	DRB1*07:01	DQB1*05:01	DQB1*02:01
2	DRB1*04:07	DRB1*13:02	DQB1*03:01	DQB1*06:04
3	DRB1*04:09	DRB1*12:01	DQB1*03:01	DQB1*03:01
4	DRB1*04:07	DRB1*15:03	DQB1*03:02	DQB1*06:02
5	DRB1*13:01	DRB1*15:01	DQB1*06:03	DQB1*06:02
6	DRB1*15:01	DRB1*11:01	DQB1*06:02	NA
7	DRB1*03:02	DRB1*15:03	DQB1*02:01	DQB1*06:02
8	DRB1*11:01	DRB1*11:01	DQB1*03:01	DQB1*03:01
9	DRB1*01:01	DRB1*11:01	DQB1*05:01	DQB1*03:01
10	DRB1*07:01	DRB1*16:02	DQB1*02:01	DQB1*03:01
11	DRB1*07:01	DRB1*11:04	DQB1*02:01	DQB1*03:01
12	DRB1*13:03	DRB1*15:02	DQB1*03:01	DQB1*06:01
13	DRB1*04:04	DRB1*15:01	DQB1*03:02	DQB1*06:02
14	DRB1*04:04	DRB1*15:01	DQB1*03:02	DQB1*06:02
15	DRB1*03:01	DRB1*11:01	DQB1*02:01	DQB1*03:01
16	DRB1*03:01	DRB1*15:01	DQB1*02:01	DQB1*06:02
17	DRB1*07:01	DRB1*11:01	DQB1*02:01	DQB1*03:01
18	DRB1*01:02	DRB1*15:03	DQB1*05:01	DQB1*06:02
19	DRB1*03:01	DRB1*13:02	DQB1*02:01	DQB1*06:04
20	DRB1*01:01	DRB1*10:01	DQB1*05:01	DQB1*05:01
21	DRB1*11:01	DRB1*13:01	DQB1*03:01	DQB1*06:03
22	DRB1*04:08	DRB1*07:01	DQB1*03:01	DQB1*30:32
23	DRB1*07:01	DRB1*13:01	DQB1*02:01	DQB1*06:03
24	DRB1*04:01	DRB1*07:01	DQB1*03:02	DQB1*02:01
25	DRB1*08:02	NA	DQB1*04:02	DQB1*05:03
26	DRB1*03:02	DRB1*15:01	DQB1*04:02	DQB1*06:02
27	DRB1*03:01	DRB1*13:01	DQB1*02:01	DQB1*06:03
28	DRB1*15:03	DRB1*07:01	DQB1*06:02	DQB1*02:01
29	DRB1*01:01	DRB1*07:01	DQB1*05:01	DQB1*02:01
30	DRB1*11:04	DRB1*16:01	DQB1*03:01	DQB1*05:02
31	DRB1*04:05	DRB1*07:01	DQB1*02:01	DQB1*02:01
32	DRB1*13:03	DRB1*15:01	DQB1*03:01	DQB1*06:02
33	DRB1*13:03	DRB1*07:01	DQB1*03:01	DQB1*02:01
34	DRB1*11:01	DRB1*14:02	DQB1*03:01	DQB1*03:01
35	DRB1*04:05	DRB1*13:02	DQB1*03:02	DQB1*06:04
36	DRB1*11:01	DRB1*15:01	DQB1*03:01	DQB1*06:02
37	DRB1*04:11	DRB1*13:02	DQB1*03:02	NA
38	DRB1*03:01	DRB1*08:02	DQB1*02:01	DQB1*04:02
39	DRB1*04:05	DRB1*11:04	DQB1*03:02	DQB1*03:01
40	DRB1*03:02	DRB1*07:01	DQB1*04:02	DQB1*02:01
41	DRB1*08:07	DRB1*14:01	DQB1*04:02	DQB1*06:03
42	DRB1*01:01	DRB1*04:05	DQB1*05:01	DQB1*03:02
43	DRB1*08:02	DRB1*13:01	DQB1*04:02	DQB1*06:03
44	DRB1*08:07	DRB1*11:01	DQB1*04:02	DQB1*03:01

45	DRB1*15:01	DRB1*04:04	DQB1*06:02	DQB1*04:02
46	DRB1*04:07	DRB1*08:01	DQB1*03:02	DQB1*04:02
47	DRB1*14:01	DRB1*16:02	DQB1*05:03	DQB1*03:01
48	DRB1*07:01	DRB1*10:01	DQB1*02:01	DQB1*05:01
49	DRB1*08:02	DRB1*13:01	DQB1*04:02	DQB1*06:03
50	DRB1*01:02	DRB1*03:02	DQB1*05:01	DQB1*04:02
51	DRB1*03:02	DRB1*03:02	DQB1*04:02	DQB1*04:02
52	DRB1*15:03	DRB1*15:03	DQB1*06:02	DQB1*06:02
53	DRB1*01:01	DRB1*11:01	DQB1*05:01	DQB1*03:01
54	DRB1*03:01	DRB1*08:02	DQB1*02:01	NA
55	DRB1*04:05	DRB1*07:01	DQB1*03:02	DQB1*02:01
56	DRB1*04:01	DRB1*04:05	DQB1*03:02	DQB1*03:02
57	DRB1*15:01	DRB1*14:01	DQB1*05:02	DQB1*05:03
58	DRB1*04:04	DRB1*11:01	DQB1*03:02	DQB1*06:02
59	DRB1*04:03	DRB1*12:01	DQB1*03:02	DQB1*05:01
60	DRB1*13:01	DRB1*13:01	DQB1*06:03	DQB1*06:03
61	DRB1*03:01	DRB1*04:11	DQB1*02:01	DQB1*03:02
62	DRB1*08:01	DRB1*14:02	DQB1*04:02	DQB1*03:01
63	DRB1*11:04	DRB1*15:01	DQB1*03:01	DQB1*05:02
64	DRB1*04:04	DRB1*08:04	DQB1*03:02	DQB1*03:01
65	DRB1*13:02	DRB1*15:01	DQB1*06:04	DQB1*06:02
66	DRB1*04:04	DRB1*11:04	DQB1*03:02	DQB1*03:01
67	DRB1*15:01	DRB1*04:04	DQB1*06:03	DQB1*03:02
68	DRB1*15:01	DRB1*04:07	DQB1*06:02	DQB1*03:01
69	DRB1*08:07	DRB1*13:01	DQB1*04:02	DQB1*06:04
70	DRB1*01:02	DRB1*07:01	DQB1*05:01	DQB1*02:01
71	DRB1*07:01	DRB1*15:01	DQB1*02:01	DQB1*06:02
72	DRB1*08:01	DRB1*14:02	DQB1*04:02	DQB1*03:01
73	DRB1*01:02	DRB1*08:02	DQB1*05:01	DQB1*04:02
74	DRB1*08:01	DRB1*11:04	DQB1*06:02	DQB1*06:02
75	DRB1*13:03	DRB1*15:01	DQB1*03:01	DQB1*06:02
76	DRB1*04:05	DRB1*11:01	DQB1*03:02	DQB1*03:01
77	DRB1*01:01	DRB1*04:06	DQB1*05:01	DQB1*04:02
78	DRB1*11:01	DRB1*16:01	DQB1*06:02	DQB1*05:02
79	DRB1*03:01	DRB1*13:01	DQB1*02:01	DQB1*06:03
80	DRB1*04:11	DRB1*13:01	DQB1*03:02	DQB1*06:04
81	DRB1*13:03	DRB1*07:01	DQB1*03:01	DQB1*02:01
82	DRB1*03:01	DRB1*08:03	DQB1*02:01	DQB1*03:01
83	DRB1*07:01	DRB1*15:01	DQB1*02:01	DQB1*06:02
84	DRB1*01:02	DRB1*08:01	DQB1*05:01	DQB1*05:01
85	DRB1*03:01	DRB1*15:01	DQB1*02:01	DQB1*06:02
86	DRB1*15:03	DRB1*14:02	DQB1*06:02	DQB1*03:01
87	DRB1*08:04	DRB1*15:03	DQB1*03:01	DQB1*06:02
88	DRB1*15:03	DRB1*15:03	DQB1*06:02	DQB1*06:02
89	DRB1*07:01	DRB1*07:01	DQB1*02:01	DQB1*30:32
90	DRB1*01:01	DRB1*04:05	DQB1*05:01	DQB1*03:02
91	DRB1*07:01	DRB1*08:04	DQB1*02:01	DQB1*04:02

92	DRB1*07:01	DRB1*07:01	DQB1*02:01	DQB1*02:01
93	DRB1*09:01	DRB1*16:01	DQB1*30:32	DQB1*05:02
94	DRB1*01:02	DRB1*14:02	DQB1*05:01	DQB1*03:01
95	DRB1*11:04	DRB1*13:01	DQB1*03:01	DQB1*05:01
96	DRB1*04:11	DRB1*07:01	DQB1*03:02	DQB1*02:01
97	DRB1*13:01	DRB1*15:01	DQB1*06:03	DQB1*06:02
98	DRB1*03:01	DRB1*07:01	DQB1*02:01	DQB1*02:01
99	DRB1*01:02	DRB1*15:03	DQB1*05:01	DQB1*06:02
100	DRB1*01:02	DRB1*03:01	DQB1*05:01	DQB1*02:01
101	DRB1*04:11	DRB1*11:01	DQB1*03:02	DQB1*03:01
102	DRB1*13:02	NA	DQB1*06:04	NA
103	NA	NA	DQB1*03:02	NA
104	DRB1*15:01	DRB1*15:03	DQB1*06:02	DQB1*06:02
105	DRB1*04:02	DRB1*14:02	NA	NA
106	DRB1*03:01	DRB1*15:01	DQB1*02:01	DQB1*06:02
107	DRB1*01:02	DRB1*03:01	DQB1*05:01	DQB1*02:01
108	DRB1*11:01	DRB1*13:03	DQB1*03:01	DQB1*03:01
109	DRB1*04:11	DRB1*11:04	DQB1*03:02	DQB1*03:01
110	DRB1*07:01	DRB1*15:01	DQB1*30:32	DQB1*06:02
111	DRB1*10:01	DRB1*15:01	DQB1*05:01	DQB1*06:02
112	DRB1*03:01	DRB1*13:01	DQB1*02:01	DQB1*06:03
113	DRB1*01:01	DRB1*08:01	DQB1*05:01	DQB1*04:02
114	DRB1*07:01	DRB1*09:01	DQB1*02:01	DQB1*30:32
115	DRB1*08:01	DRB1*14:01	DQB1*04:02	DQB1*05:03
116	DRB1*03:01	DRB1*08:07	DQB1*02:01	DQB1*04:02
117	DRB1*11:02	DRB1*16:02	DQB1*03:01	DQB1*03:01
118	DRB1*03:01	DRB1*09:01	DQB1*02:01	DQB1*30:32
119	DRB1*01:02	DRB1*13:01	DQB1*05:01	DQB1*06:03
120	DRB1*08:02	DRB1*11:02	DQB1*04:02	DQB1*03:01
121	DRB1*04:05	DRB1*07:01	DQB1*02:01	DQB1*02:01
122	DRB1*11:04	DRB1*14:01	DQB1*03:01	DQB1*05:03
123	DRB1*07:01	DRB1*15:01	DQB1*02:01	DQB1*06:02
124	DRB1*04:03	DRB1*04:03	DQB1*03:01	DQB1*03:01
125	DRB1*07:01	DRB1*15:03	DQB1*02:01	DQB1*06:02
126	DRB1*10:01	DRB1*10:01	DQB1*05:01	DQB1*05:01
127	DRB1*04:05	DRB1*09:01	DQB1*03:02	DQB1*30:32
128	DRB1*07:01	DRB1*15:03	DQB1*02:01	DQB1*06:02
129	DRB1*13:02	DRB1*16:02	DQB1*05:02	DQB1*03:01
130	NA	NA	DQB1*05:01	DQB1*03:01
131	DRB1*15:03	DRB1*11:01	DQB1*06:02	NA
132	NA	DRB1*13:01	DQB1*05:01	DQB1*06:04
133	DRB1*11:04	DRB1*11:04	DQB1*03:01	DQB1*03:01
134	DRB1*01:02	DRB1*11:01	DQB1*05:01	DQB1*06:02
135	DRB1*10:01	DRB1*07:01	DQB1*05:01	DQB1*02:01
136	DRB1*13:40	DRB1*16:02	DQB1*06:03	DQB1*05:02
137	DRB1*07:01	DRB1*10:01	DQB1*02:01	DQB1*05:01
138	DRB1*11:01	DRB1*11:01	DQB1*03:01	DQB1*03:01

139	DRB1*03:01	DRB1*11:01	DQB1*02:01	DQB1*03:01
140	DRB1*04:04	DRB1*10:01	DQB1*03:02	DQB1*05:01
141	DRB1*03:01	DRB1*03:01	DQB1*02:01	DQB1*02:01
142	DRB1*01:02	DRB1*04:04	DQB1*05:01	DQB1*03:02
143	DRB1*01:01	DRB1*12:01	DQB1*05:01	DQB1*05:01
144	DRB1*16:02	DRB1*11:02	DQB1*05:02	DQB1*03:01
145	DRB1*01:01	DRB1*09:01	DQB1*05:01	DQB1*02:01
146	DRB1*08:07	DRB1*11:01	DQB1*04:02	DQB1*03:01
147	DRB1*04:11	DRB1*15:01	DQB1*03:02	DQB1*06:02
148	DRB1*11:01	DRB1*13:02	DQB1*03:01	DQB1*06:04
149	DRB1*11:01	DRB1*13:01	DQB1*03:01	DQB1*06:03
150	DRB1*01:01	DRB1*07:01	DQB1*05:01	DQB1*02:01
151	DRB1*03:02	DRB1*11:02	DQB1*04:02	DQB1*03:01
152	DRB1*13:01	NA	DQB1*06:03	DQB1*06:04
153	DRB1*04:04	DRB1*16:02	DQB1*03:02	DQB1*03:01
154	DRB1*03:01	DRB1*09:01	DQB1*02:01	DQB1*30:32
155	DRB1*14:01	NA	DQB1*05:03	DQB1*06:04
156	DRB1*10:01	DRB1*15:03	DQB1*05:01	DQB1*06:02
157	DRB1*11:01	NA	DQB1*03:01	DQB1*06:02
158	DRB1*11:01	DRB1*15:03	DQB1*03:01	DQB1*06:02
159	DRB1*04:03	DRB1*07:01	DQB1*03:02	DQB1*02:01
160	DRB1*03:01	DRB1*11:01	DQB1*02:01	DQB1*06:02
161	DRB1*08:02	DRB1*13:01	DQB1*04:02	DQB1*06:03
162	DRB1*07:01	DRB1*09:01	DQB1*02:01	DQB1*30:32
163	DRB1*13:01	DRB1*13:01	DQB1*06:03	DQB1*06:03
164	DRB1*15:01	DRB1*15:03	DQB1*05:02	DQB1*06:02
165	DRB1*11:01	DRB1*11:02	DQB1*06:02	DQB1*03:01
166	DRB1*15:01	DRB1*15:03	DQB1*06:02	DQB1*06:02
167	DRB1*03:02	DRB1*14:01	DQB1*04:02	DQB1*05:03
168	DRB1*04:03	DRB1*16:02	DQB1*03:02	DQB1*05:02
169	DRB1*16:02	DRB1*16:02	DQB1*03:01	DQB1*03:01
170	DRB1*04:08	DRB1*14:01	DQB1*03:01	DQB1*05:03
171	DRB1*01:01	DRB1*13:01	DQB1*05:01	DQB1*06:03
172	DRB1*15:03	DRB1*07:01	DQB1*06:02	DQB1*02:01
173	DRB1*11:04	DRB1*13:02	DQB1*03:01	DQB1*06:01
174	DRB1*04:03	DRB1*13:02	DQB1*03:02	DQB1*06:04
175	DRB1*13:01	DRB1*15:03	DQB1*06:03	DQB1*06:02
176	DRB1*09:01	DRB1*11:01	DQB1*02:01	DQB1*03:01
177	DRB1*01:02	DRB1*09:01	DQB1*05:01	DQB1*02:01
178	DRB1*03:02	DRB1*13:03	DQB1*04:02	DQB1*03:01
179	DRB1*15:01	DRB1*15:01	DQB1*06:02	DQB1*06:02
180	DRB1*03:02	DRB1*16:02	DQB1*04:02	DQB1*05:02
181	DRB1*03:01	DRB1*07:01	DQB1*02:01	DQB1*02:01
182	DRB1*03:01	DRB1*03:01	DQB1*02:01	DQB1*02:01
183	DRB1*14:01	DRB1*07:01	DQB1*05:03	DQB1*30:32
184	DRB1*01:02	DRB1*11:02	DQB1*05:01	DQB1*03:01
185	DRB1*11:01	DRB1*13:04	NA	DQB1*03:01

186	DRB1*13:01	DRB1*10:01	DQB1*06:03	DQB1*05:01
187	DRB1*01:01	DRB1*15:03	DQB1*05:01	DQB1*06:02
188	DRB1*15:01	DRB1*03:01	DQB1*06:02	DQB1*02:01
189	DRB1*11:02	DRB1*09:01	DQB1*03:01	DQB1*02:01
190	DRB1*07:01	DRB1*11:01	DQB1*02:01	DQB1*03:01
191	DRB1*13:01	DRB1*13:01	DQB1*05:01	DQB1*06:02
192	DRB1*03:01	DRB1*14:02	DQB1*02:01	DQB1*03:01
193	DRB1*04:11	DRB1*07:01	DQB1*03:02	DQB1*02:01
194	DRB1*07:01	DRB1*15:01	DQB1*02:01	DQB1*06:02
195	DRB1*09:01	DRB1*11:04	DQB1*02:01	DQB1*03:01
196	DRB1*01:02	DRB1*14:01	DQB1*05:01	NA
197	DRB1*08:07	DRB1*16:02	DQB1*03:01	DQB1*03:01
198	DRB1*07:01	DRB1*11:01	DQB1*02:01	DQB1*03:01
199	DRB1*08:06	DRB1*11:04	DQB1*03:01	DQB1*06:02
200	DRB1*11:01	DRB1*11:01	DQB1*03:01	DQB1*06:02
201	DRB1*11:01	DRB1*11:01	DQB1*03:01	DQB1*05:02
202	DRB1*10:01	DRB1*13:01	DQB1*03:01	DQB1*06:03
203	NA	NA	NA	DQB1*03:01
204	NA	NA	DQB1*03:02	DQB1*03:02
205	DRB1*11:03	DRB1*13:01	DQB1*03:01	DQB1*06:03
206	DRB1*07:01	DRB1*11:01	DQB1*02:01	DQB1*03:01
207	DRB1*03:01	DRB1*08:07	DQB1*02:01	DQB1*04:02
208	DRB1*03:01	DRB1*07:01	DQB1*02:01	DQB1*02:01
209	DRB1*10:01	DRB1*11:04	DQB1*05:01	DQB1*05:02
210	DRB1*13:02	DRB1*13:02	DQB1*05:01	DQB1*06:05
211	DRB1*01:02	DRB1*07:01	DQB1*05:01	DQB1*30:32
212	DRB1*07:01	DRB1*08:07	DQB1*02:01	DQB1*04:02
213	DRB1*01:02	DRB1*11:01	DQB1*05:01	DQB1*03:01
214	DRB1*12:01	DRB1*16:02	DQB1*05:01	DQB1*03:01
215	DRB1*08:04	DRB1*15:03	DQB1*03:01	DQB1*06:02
216	DRB1*14:01	DRB1*16:02	DQB1*05:03	DQB1*03:01
217	DRB1*03:01	DRB1*10:01	DQB1*02:01	DQB1*05:01
218	DRB1*01:01	DRB1*15:01	DQB1*05:01	DQB1*06:02
219	DRB1*01:02	DRB1*11:02	DQB1*05:01	NA
220	DRB1*01:01	DRB1*15:01	DQB1*05:01	DQB1*06:02
221	DRB1*12:01	DRB1*12:01	DQB1*05:01	DQB1*05:01
222	DRB1*10:01	NA	DQB1*05:01	DQB1*03:01
223	DRB1*04:01	NA	DQB1*03:02	DQB1*03:01
224	DRB1*01:01	DRB1*07:01	DQB1*05:01	DQB1*02:01
225	DRB1*04:04	DRB1*08:01	DQB1*03:02	DQB1*04:02
226	DRB1*15:01	NA	DQB1*06:02	DQB1*03:01
227	DRB1*04:05	DRB1*15:01	DQB1*03:02	DQB1*06:02
228	DRB1*03:01	DRB1*07:01	DQB1*02:01	DQB1*02:01
229	DRB1*11:01	DRB1*10:01	DQB1*06:02	DQB1*05:01
230	DRB1*13:01	DRB1*15:01	DQB1*06:03	DQB1*06:02
231	DRB1*15:01	DRB1*13:01	DQB1*06:02	DQB1*05:01
232	DRB1*03:01	DRB1*13:03	DQB1*02:01	DQB1*03:01

233	DRB1*15:01	DRB1*14:01	DQB1*06:02	DQB1*05:03
234	DRB1*03:01	DRB1*11:01	DQB1*02:01	DQB1*03:01
235	DRB1*04:05	DRB1*07:01	DQB1*03:02	DQB1*02:01
236	DRB1*01:02	DRB1*13:02	DQB1*05:01	NA
237	DRB1*10:01	DRB1*13:02	DQB1*30:32	DQB1*06:05
238	DRB1*11:01	DRB1*13:02	DQB1*06:02	DQB1*05:02
239	DRB1*01:02	DRB1*03:01	DQB1*05:01	DQB1*02:01
240	DRB1*10:01	DRB1*15:01	DQB1*05:01	DQB1*06:02
241	DRB1*01:02	DRB1*03:02	DQB1*05:01	DQB1*04:02
242	DRB1*11:01	DRB1*13:02	DQB1*06:02	DQB1*06:05
243	DRB1*01:02	DRB1*13:03	DQB1*05:01	NA
244	DRB1*01:02	DRB1*03:02	DQB1*05:01	DQB1*04:02
245	DRB1*07:01	DRB1*07:01	DQB1*02:01	DQB1*02:01
246	DRB1*03:01	DRB1*13:01	DQB1*02:01	NA
247	DRB1*01:01	DRB1*14:01	DQB1*05:01	DQB1*05:03
248	DRB1*07:01	DRB1*13:01	DQB1*02:01	DQB1*06:03
249	DRB1*01:02	DRB1*08:04	DQB1*05:01	DQB1*03:01
250	DRB1*11:01	NA	DQB1*06:02	DQB1*30:32
251	DRB1*13:01	DRB1*14:06	DQB1*06:03	DQB1*03:01
252	DRB1*01:01	DRB1*13:01	DQB1*05:01	DQB1*06:03
253	DRB1*07:01	DRB1*16:02	DQB1*02:01	DQB1*03:01
254	DRB1*07:01	DRB1*11:01	DQB1*02:01	DQB1*03:01
255	DRB1*03:01	DRB1*09:01	DQB1*02:01	DQB1*02:01
256	DRB1*07:01	DRB1*13:02	DQB1*02:01	DQB1*06:04
257	DRB1*01:02	DRB1*07:01	DQB1*05:01	DQB1*02:01
258	DRB1*01:02	DRB1*10:01	DQB1*05:01	DQB1*05:01
259	DRB1*11:01	DRB1*11:02	DQB1*03:01	DQB1*03:01
260	DRB1*03:01	DRB1*07:01	DQB1*02:01	DQB1*30:32
261	DRB1*07:01	DRB1*15:01	DQB1*02:01	DQB1*06:02
262	DRB1*01:02	DRB1*04:03	DQB1*05:01	DQB1*03:02
263	DRB1*01:02	DRB1*12:01	DQB1*05:01	DQB1*05:01
264	DRB1*07:01	DRB1*13:02	DQB1*02:01	DQB1*06:04
265	DRB1*03:01	NA	DQB1*02:01	NA
266	DRB1*03:01	DRB1*07:01	DQB1*02:01	DQB1*02:01
267	DRB1*15:01	DRB1*13:01	DQB1*06:02	DQB1*06:03
268	DRB1*04:11	DRB1*16:02	DQB1*03:02	DQB1*03:01
269	DRB1*09:01	DRB1*15:01	DQB1*02:01	DQB1*06:02
270	DRB1*13:02	DRB1*07:01	DQB1*06:04	DQB1*02:01
271	DRB1*01:03	DRB1*13:01	DQB1*05:01	DQB1*06:03
272	DRB1*09:01	DRB1*15:03	DQB1*02:01	DQB1*06:02
273	DRB1*03:01	DRB1*07:01	DQB1*02:01	DQB1*02:01
274	DRB1*03:01	DRB1*07:01	DQB1*02:01	DQB1*02:01
275	DRB1*07:01	DRB1*11:01	DQB1*02:01	DQB1*03:01
276	DRB1*03:01	DRB1*13:01	DQB1*02:01	DQB1*06:03
277	DRB1*09:01	DRB1*15:03	DQB1*02:01	DQB1*06:02
278	DRB1*07:01	DRB1*11:04	DQB1*30:32	DQB1*03:01
279	DRB1*01:02	DRB1*15:03	DQB1*05:01	DQB1*06:02

280	DRB1*01:03	DRB1*07:01	DQB1*05:01	DQB1*02:01
281	DRB1*01:01	DRB1*07:01	DQB1*05:01	DQB1*02:01
282	DRB1*15:01	DRB1*15:02	DQB1*06:02	NA
283	DRB1*04:05	NA	DQB1*02:01	DQB1*03:01
284	DRB1*01:03	DRB1*12:01	DQB1*05:01	DQB1*05:01
285	DRB1*03:01	DRB1*07:01	DQB1*02:01	DQB1*30:32
286	DRB1*01:01	DRB1*07:01	DQB1*05:01	DQB1*02:01
287	DRB1*09:01	DRB1*13:02	DQB1*30:32	DQB1*06:05
288	DRB1*15:03	DRB1*07:01	DQB1*06:02	DQB1*02:01
289	DRB1*03:02	DRB1*13:02	DQB1*04:02	DQB1*05:01
290	DRB1*15:01	DRB1*15:01	DQB1*06:02	DQB1*06:02
291	DRB1*15:01	DRB1*03:01	DQB1*06:02	DQB1*02:01
292	DRB1*15:01	DRB1*03:01	DQB1*06:03	DQB1*02:01
293	DRB1*13:02	DRB1*13:03	DQB1*06:04	DQB1*03:01
294	DRB1*01:01	DRB1*08:07	DQB1*05:01	DQB1*04:02
295	DRB1*03:01	DRB1*07:01	DQB1*02:01	DQB1*30:32
296	DRB1*03:02	DRB1*11:01	DQB1*04:02	DQB1*06:02
297	DRB1*11:02	DRB1*09:01	DQB1*03:01	DQB1*02:01
298	DRB1*11:01	DRB1*15:01	DQB1*03:01	DQB1*06:02
299	DRB1*09:01	DRB1*12:01	DQB1*02:01	DQB1*03:01
300	DRB1*13:01	DRB1*15:01	DQB1*06:03	DQB1*06:02
301	DRB1*07:01	DRB1*10:01	DQB1*02:01	DQB1*05:01
302	DRB1*01:01	DRB1*07:01	DQB1*05:01	DQB1*02:01
303	DRB1*08:01	DRB1*08:04	DQB1*04:02	DQB1*04:02
304	DRB1*07:01	DRB1*15:01	DQB1*30:32	DQB1*06:02
305	DRB1*03:01	DRB1*13:02	DQB1*02:01	DQB1*05:01
306	DRB1*15:01	NA	DQB1*06:02	DQB1*03:01
307	DRB1*07:01	DRB1*11:04	DQB1*02:01	DQB1*03:01
308	DRB1*01:02	DRB1*11:01	DQB1*05:01	DQB1*06:02
309	DRB1*04:04	DRB1*07:01	DQB1*03:02	DQB1*02:01
310	DRB1*10:01	DRB1*12:02	DQB1*05:01	DQB1*03:01
311	DRB1*13:02	DRB1*13:02	NA	DQB1*06:05
312	DRB1*01:01	DRB1*13:01	DQB1*05:01	DQB1*06:03
313	DRB1*11:03	DRB1*13:01	DQB1*03:01	DQB1*30:32
314	DRB1*01:02	DRB1*07:01	DQB1*05:01	DQB1*02:01
315	DRB1*08:01	DRB1*11:01	DQB1*04:02	DQB1*06:02
316	DRB1*01:01	DRB1*12:01	DQB1*05:01	DQB1*05:01
317	DRB1*03:01	DRB1*07:01	DQB1*02:01	DQB1*02:01
318	DRB1*04:01	DRB1*04:03	DQB1*03:01	DQB1*03:02
319	DRB1*07:01	DRB1*13:02	DQB1*02:01	DQB1*06:05
320	DRB1*01:02	DRB1*03:01	DQB1*05:01	DQB1*02:01
321	DRB1*04:05	DRB1*13:01	DQB1*03:02	DQB1*06:03
322	DRB1*04:04	DRB1*08:02	DQB1*03:02	DQB1*04:02
323	DRB1*11:01	DRB1*13:01	DQB1*03:01	DQB1*06:03
324	DRB1*07:01	DRB1*08:07	DQB1*02:01	DQB1*04:02
325	DRB1*07:01	DRB1*07:01	DQB1*02:01	DQB1*02:01
326	DRB1*08:02	DRB1*07:01	DQB1*04:02	DQB1*02:01

327	DRB1*04:05	DRB1*13:01	DQB1*02:01	DQB1*06:03
328	DRB1*07:01	DRB1*11:01	DQB1*30:32	DQB1*06:04
329	DRB1*11:01	DRB1*13:02	DQB1*03:01	DQB1*06:04
330	DRB1*01:01	NA	DQB1*05:01	DQB1*06:02
331	DRB1*03:02	DRB1*13:01	DQB1*04:02	DQB1*06:03
332	DRB1*01:02	DRB1*13:03	DQB1*05:01	NA
333	DRB1*13:02	DRB1*13:03	DQB1*06:05	DQB1*03:01
334	DRB1*03:02	DRB1*11:01	DQB1*04:02	DQB1*06:02
335	DRB1*04:01	DRB1*07:01	DQB1*03:02	DQB1*02:01
336	DRB1*13:02	DRB1*15:03	DQB1*05:01	DQB1*06:02
337	DRB1*03:01	DRB1*03:01	DQB1*02:01	DQB1*02:01
338	DRB1*01:01	DRB1*10:01	DQB1*05:01	DQB1*05:01
339	DRB1*03:01	DRB1*13:02	DQB1*02:01	DQB1*06:04
340	DRB1*08:04	DRB1*07:01	DQB1*03:01	DQB1*02:01
341	DRB1*15:01	DRB1*11:01	DQB1*06:02	DQB1*06:02
342	DRB1*07:01	DRB1*14:10	DQB1*30:32	DQB1*06:04
343	DRB1*01:01	DRB1*09:01	DQB1*05:01	DQB1*02:01
344	DRB1*07:01	DRB1*11:02	DQB1*02:01	DQB1*03:01
345	DRB1*01:01	DRB1*13:01	DQB1*05:01	DQB1*06:03
346	DRB1*11:01	DRB1*11:02	DQB1*03:01	DQB1*03:01
347	DRB1*16:01	DRB1*03:02	DQB1*05:02	DQB1*04:02
348	DRB1*16:02	DRB1*04:04	DQB1*05:02	NA
349	DRB1*01:01	DRB1*03:02	DQB1*05:01	DQB1*04:02
350	DRB1*03:01	DRB1*13:02	DQB1*02:01	DQB1*05:02
351	DRB1*08:06	DRB1*15:01	DQB1*06:02	DQB1*06:02
352	DRB1*07:01	DRB1*08:04	DQB1*02:01	DQB1*03:01
353	DRB1*11:01	DRB1*16:01	DQB1*03:01	DQB1*05:02
354	DRB1*07:01	DRB1*13:01	DQB1*02:01	DQB1*06:03
355	DRB1*03:02	DRB1*10:01	DQB1*04:02	DQB1*05:01
356	DRB1*01:01	DRB1*14:01	DQB1*05:01	DQB1*05:03
357	DRB1*07:01	DRB1*13:02	DQB1*02:01	DQB1*06:05
358	DRB1*11:04	DRB1*13:02	DQB1*03:01	DQB1*06:04
359	DRB1*03:01	DRB1*10:01	DQB1*02:01	DQB1*05:01
360	DRB1*13:01	DRB1*13:02	DQB1*06:03	DQB1*06:04
361	DRB1*01:02	DRB1*16:02	DQB1*05:01	NA
362	DRB1*08:01	DRB1*13:01	DQB1*04:02	DQB1*06:03
363	DRB1*04:04	DRB1*08:04	DQB1*03:02	DQB1*03:01
364	DRB1*07:01	DRB1*15:03	DQB1*02:01	DQB1*06:02
365	DRB1*16:02	DRB1*11:01	DQB1*03:01	DQB1*03:01
366	DRB1*04:06	DRB1*07:01	DQB1*04:02	DQB1*30:32
367	DRB1*15:03	DRB1*16:01	DQB1*06:02	DQB1*05:02
368	DRB1*03:01	DRB1*08:02	DQB1*02:01	DQB1*04:02
369	DRB1*11:01	DRB1*07:01	NA	DQB1*02:01
370	DRB1*04:11	DRB1*07:01	DQB1*03:02	DQB1*02:01
371	DRB1*01:02	DRB1*04:01	DQB1*05:01	DQB1*03:02
372	DRB1*11:01	DRB1*11:01	DQB1*03:01	DQB1*03:01
373	DRB1*03:01	DRB1*11:01	DQB1*02:01	DQB1*03:01

374	DRB1*13:01	DRB1*07:01	DQB1*06:03	DQB1*02:01
375	DRB1*04:05	DRB1*11:03	DQB1*03:02	DQB1*03:01
376	DRB1*07:01	DRB1*09:01	DQB1*02:01	DQB1*02:01
377	DRB1*03:01	DRB1*15:01	DQB1*02:01	DQB1*06:02
378	DRB1*07:01	DRB1*16:01	DQB1*02:01	DQB1*05:02
379	DRB1*01:01	DRB1*03:01	DQB1*05:01	DQB1*02:01
380	DRB1*11:01	DRB1*11:02	DQB1*06:02	DQB1*03:01
381	DRB1*04:05	DRB1*07:01	DQB1*03:02	DQB1*30:32
382	DRB1*15:01	DRB1*04:05	DQB1*06:02	DQB1*30:32
383	DRB1*07:01	DRB1*13:02	DQB1*02:01	DQB1*06:04
384	DRB1*11:01	DRB1*11:01	DQB1*03:01	DQB1*03:01
385	DRB1*04:02	DRB1*08:03	DQB1*03:02	DQB1*03:01
386	DRB1*15:03	DRB1*13:01	DQB1*06:02	DQB1*06:02
387	DRB1*14:02	DRB1*16:02	DQB1*03:01	DQB1*03:01
388	DRB1*01:01	DRB1*07:01	DQB1*05:01	DQB1*02:01
389	DRB1*07:01	DRB1*13:02	DQB1*02:01	DQB1*06:05
390	DRB1*04:03	DRB1*04:05	DQB1*03:02	DQB1*30:32
391	DRB1*13:01	DRB1*13:02	DQB1*06:03	DQB1*06:04
392	DRB1*11:01	DRB1*13:01	DQB1*06:02	DQB1*06:03
393	DRB1*08:07	DRB1*15:01	DQB1*04:02	DQB1*06:02
394	DRB1*13:01	DRB1*13:01	DQB1*03:01	DQB1*05:02
395	DRB1*04:07	DRB1*08:01	DQB1*03:02	DQB1*04:02
396	DRB1*07:01	DRB1*07:01	DQB1*02:01	DQB1*02:01
397	DRB1*03:01	DRB1*11:01	DQB1*02:01	DQB1*03:01
398	DRB1*08:04	DRB1*10:01	DQB1*03:01	DQB1*05:01
399	DRB1*03:01	DRB1*07:01	DQB1*02:01	DQB1*02:01
400	DRB1*14:04	DRB1*08:04	DQB1*05:03	DQB1*03:01
401	DRB1*01:02	DRB1*03:02	DQB1*05:01	DQB1*04:02
402	DRB1*03:01	DRB1*11:02	DQB1*02:01	DQB1*03:01
403	DRB1*04:11	DRB1*07:01	DQB1*03:02	DQB1*02:01
404	DRB1*07:01	DRB1*13:02	DQB1*02:01	DQB1*06:04
405	DRB1*03:02	DRB1*11:02	DQB1*04:02	DQB1*03:01
406	DRB1*13:01	DRB1*07:01	DQB1*06:03	DQB1*02:01
407	DRB1*11:01	DRB1*07:01	DQB1*03:01	DQB1*02:01
408	DRB1*11:01	DRB1*16:01	DQB1*03:01	DQB1*05:02
409	DRB1*07:01	DRB1*13:03	DQB1*02:01	DQB1*03:01
410	DRB1*03:01	DRB1*07:01	DQB1*02:01	DQB1*02:01
411	DRB1*04:03	DRB1*07:01	DQB1*03:04	DQB1*02:01
412	DRB1*13:01	DRB1*15:01	DQB1*06:03	DQB1*06:02
413	DRB1*08:02	DRB1*15:03	DQB1*04:02	DQB1*06:02
414	DRB1*01:01	DRB1*16:02	DQB1*05:01	DQB1*05:02
415	DRB1*01:02	DRB1*03:01	DQB1*05:01	DQB1*02:01
416	DRB1*07:01	DRB1*15:01	DQB1*02:01	DQB1*06:02
417	DRB1*01:02	DRB1*03:01	DQB1*05:01	DQB1*02:01
418	DRB1*04:01	DRB1*11:01	DQB1*03:02	DQB1*03:01
419	DRB1*04:02	DRB1*08:01	DQB1*03:02	DQB1*04:02
420	DRB1*11:04	DRB1*16:01	DQB1*03:01	DQB1*05:02

421	DRB1*12:01	DRB1*14:06	DQB1*03:01	DQB1*03:01
422	DRB1*01:01	DRB1*01:01	DQB1*05:01	DQB1*05:01
423	DRB1*15:01	DRB1*10:01	DQB1*06:02	DQB1*05:01
424	DRB1*03:02	DRB1*07:01	DQB1*04:02	DQB1*02:01
425	DRB1*01:01	DRB1*15:03	DQB1*05:01	DQB1*06:02
426	DRB1*03:01	DRB1*11:01	DQB1*02:01	DQB1*03:01
427	DRB1*01:02	DRB1*03:01	DQB1*05:01	DQB1*02:01
428	DRB1*08:04	DRB1*13:02	DQB1*04:02	DQB1*06:04
429	DRB1*03:02	DRB1*07:01	DQB1*04:02	DQB1*02:01
430	DRB1*04:05	DRB1*11:01	DQB1*03:02	DQB1*03:01
431	DRB1*07:01	DRB1*11:02	DQB1*02:01	DQB1*03:01
432	DRB1*04:04	DRB1*07:01	DQB1*03:02	DQB1*02:01
433	DRB1*01:01	DRB1*03:01	DQB1*05:01	DQB1*02:01
434	DRB1*03:01	DRB1*11:01	DQB1*02:01	DQB1*03:01
435	DRB1*01:01	DRB1*01:01	DQB1*05:01	DQB1*05:01
436	DRB1*03:01	DRB1*10:01	DQB1*02:01	DQB1*05:01
437	DRB1*03:01	DRB1*07:01	DQB1*02:01	DQB1*02:01
438	DRB1*01:02	DRB1*11:02	DQB1*05:01	DQB1*03:01
439	DRB1*01:02	NA	DQB1*05:01	DQB1*03:01
440	DRB1*04:11	DRB1*11:01	DQB1*03:02	DQB1*03:01
441	DRB1*13:01	DRB1*07:01	DQB1*06:03	DQB1*02:01
442	DRB1*08:02	DRB1*13:02	DQB1*04:02	DQB1*05:01
443	DRB1*03:01	DRB1*11:01	DQB1*02:01	NA
444	DRB1*04:04	DRB1*12:01	DQB1*03:02	DQB1*03:01
445	DRB1*13:01	DRB1*07:01	DQB1*06:03	DQB1*02:01
446	DRB1*13:01	DRB1*15:03	DQB1*05:01	DQB1*06:02
447	DRB1*03:01	DRB1*04:04	DQB1*02:01	DQB1*04:02
448	DRB1*03:01	DRB1*16:01	DQB1*02:01	DQB1*05:02
449	DRB1*04:02	DRB1*09:01	DQB1*03:02	DQB1*30:32
450	DRB1*11:01	DRB1*16:02	DQB1*06:02	DQB1*03:01
451	DRB1*04:01	DRB1*07:01	DQB1*03:01	DQB1*02:01
452	DRB1*03:01	DRB1*16:02	DQB1*02:01	DQB1*03:01
453	DRB1*11:01	DRB1*11:02	DQB1*06:02	DQB1*03:01
454	DRB1*15:03	DRB1*16:02	DQB1*06:02	DQB1*03:01
455	DRB1*01:02	DRB1*04:11	DQB1*05:01	DQB1*03:02
456	DRB1*11:01	DRB1*07:01	DQB1*03:01	DQB1*02:01
457	DRB1*15:03	DRB1*07:01	DQB1*06:02	DQB1*02:01
458	DRB1*13:03	DRB1*15:01	DQB1*02:01	DQB1*06:02
459	DRB1*13:02	DRB1*14:01	DQB1*06:04	DQB1*05:03
460	DRB1*01:03	DRB1*15:01	DQB1*05:01	DQB1*06:02
461	DRB1*07:01	DRB1*07:01	DQB1*02:01	DQB1*30:32
462	DRB1*01:01	DRB1*11:02	DQB1*05:01	DQB1*03:01
463	DRB1*15:03	DRB1*15:03	DQB1*05:01	DQB1*06:02
464	DRB1*13:02	DRB1*15:03	DQB1*06:04	DQB1*06:02
465	DRB1*04:05	DRB1*07:01	DQB1*03:01	DQB1*02:01
466	DRB1*07:01	DRB1*14:01	DQB1*02:01	DQB1*05:03
467	DRB1*04:11	DRB1*13:02	DQB1*03:02	DQB1*06:05

468	DRB1*15:03	DRB1*11:03	DQB1*06:02	DQB1*03:01
469	DRB1*04:03	DRB1*10:01	DQB1*03:02	DQB1*05:01
470	DRB1*04:04	DRB1*13:01	DQB1*03:02	DQB1*06:03
471	DRB1*03:01	DRB1*15:03	DQB1*02:01	DQB1*06:02
472	DRB1*01:01	DRB1*13:01	DQB1*05:01	DQB1*06:03
473	DRB1*01:01	DRB1*14:02	DQB1*05:01	DQB1*03:01
474	DRB1*11:04	DRB1*14:01	DQB1*03:01	DQB1*05:03
475	DRB1*04:07	DRB1*08:03	DQB1*03:01	NA
476	DRB1*08:04	DRB1*15:01	DQB1*03:01	DQB1*06:02
477	DRB1*04:03	DRB1*07:01	DQB1*03:02	DQB1*02:01
478	DRB1*04:01	DRB1*11:01	DQB1*03:02	DQB1*03:01
479	DRB1*08:07	DRB1*09:01	DQB1*04:02	DQB1*30:32
480	DRB1*08:01	DRB1*11:04	DQB1*05:02	DQB1*06:02
481	DRB1*03:02	DRB1*07:01	DQB1*04:02	DQB1*02:01
482	DRB1*07:01	DRB1*07:01	DQB1*02:01	DQB1*02:01
483	DRB1*11:01	DRB1*13:01	DQB1*03:01	DQB1*06:03
484	DRB1*04:07	DRB1*13:01	DQB1*03:02	DQB1*05:01
485	DRB1*10:01	DRB1*13:01	DQB1*30:32	DQB1*05:01
486	DRB1*15:03	DRB1*15:03	DQB1*06:02	DQB1*06:02
487	DRB1*15:01	DRB1*15:01	DQB1*06:02	DQB1*06:02
488	DRB1*07:01	DRB1*11:01	DQB1*02:01	DQB1*04:02
489	DRB1*14:01	DRB1*15:01	DQB1*05:03	DQB1*06:02
490	DRB1*11:01	DRB1*13:02	DQB1*06:02	DQB1*06:05
491	DRB1*09:01	DRB1*13:02	DQB1*02:01	DQB1*06:05
492	DRB1*01:01	DRB1*16:02	DQB1*05:01	DQB1*03:01
493	DRB1*13:01	DRB1*16:01	DQB1*06:03	DQB1*05:02
494	DRB1*03:02	DRB1*14:01	DQB1*04:02	DQB1*05:01
495	DRB1*08:01	DRB1*07:01	DQB1*04:02	DQB1*30:32
496	DRB1*03:01	DRB1*07:01	DQB1*02:01	DQB1*02:01
497	DRB1*01:01	DRB1*13:01	DQB1*05:01	DQB1*06:03
498	DRB1*07:01	DRB1*08:03	DQB1*02:01	DQB1*03:01
499	DRB1*03:01	DRB1*10:01	DQB1*02:01	NA
500	DRB1*01:01	DRB1*13:02	DQB1*05:01	DQB1*06:05
501	DRB1*04:02	DRB1*03:02	DQB1*03:02	DQB1*04:02
502	DRB1*09:01	DRB1*12:01	DQB1*02:01	DQB1*03:01
503	DRB1*07:01	DRB1*11:04	DQB1*02:01	DQB1*03:01
504	DRB1*01:01	DRB1*09:01	DQB1*05:01	DQB1*30:32
505	DRB1*09:01	DRB1*07:01	NA	DQB1*02:01
506	DRB1*10:01	DRB1*11:02	DQB1*05:01	DQB1*03:01
507	DRB1*03:01	DRB1*11:03	DQB1*02:01	DQB1*03:01
508	DRB1*16:02	DRB1*14:06	NA	NA
509	DRB1*03:01	DRB1*11:01	DQB1*02:01	DQB1*03:01
510	DRB1*03:02	DRB1*04:07	DQB1*04:02	DQB1*03:02
511	DRB1*15:03	NA	DQB1*06:02	DQB1*03:01
512	DRB1*07:01	DRB1*13:01	DQB1*02:01	DQB1*06:03
513	DRB1*01:01	DRB1*08:01	DQB1*05:01	DQB1*04:02
514	DRB1*04:02	DRB1*04:05	DQB1*03:02	DQB1*30:32

515	DRB1*01:01	DRB1*15:03	DQB1*05:01	DQB1*06:02
516	DRB1*01:01	NA	DQB1*05:01	NA
517	DRB1*03:01	DRB1*08:04	DQB1*02:01	DQB1*03:01
518	DRB1*01:01	DRB1*15:03	DQB1*05:01	DQB1*06:02
519	DRB1*13:01	DRB1*07:01	DQB1*06:03	DQB1*02:01
520	DRB1*03:02	DRB1*13:02	DQB1*04:02	DQB1*06:04
521	DRB1*11:04	DRB1*15:01	DQB1*03:01	DQB1*06:02
522	NA	DRB1*07:01	NA	DQB1*02:01
523	DRB1*07:01	DRB1*12:01	DQB1*02:01	DQB1*03:01
524	DRB1*15:01	DRB1*16:02	DQB1*06:03	DQB1*03:01
525	DRB1*03:01	DRB1*03:01	DQB1*02:01	DQB1*02:01
526	DRB1*07:01	DRB1*11:04	DQB1*02:01	DQB1*03:01
527	DRB1*03:01	DRB1*07:01	DQB1*02:01	DQB1*02:01
528	DRB1*08:03	DRB1*11:01	DQB1*03:01	DQB1*06:02
529	NA	NA	DQB1*03:02	DQB1*30:32
530	DRB1*01:02	DRB1*13:04	DQB1*05:01	DQB1*03:01
531	DRB1*07:01	DRB1*08:07	DQB1*02:01	DQB1*04:02
532	DRB1*13:02	DRB1*13:02	DQB1*06:04	DQB1*06:05
533	DRB1*04:11	DRB1*15:03	DQB1*03:02	DQB1*06:02
534	DRB1*04:02	DRB1*07:01	DQB1*03:02	DQB1*02:01
535	DRB1*08:03	DRB1*11:02	DQB1*04:02	DQB1*03:01
536	DRB1*03:01	DRB1*11:01	DQB1*02:01	DQB1*06:02
537	DRB1*01:01	DRB1*08:02	DQB1*05:01	DQB1*04:02
538	DRB1*08:02	DRB1*13:02	DQB1*04:02	DQB1*06:05
539	DRB1*01:02	DRB1*15:01	DQB1*05:01	DQB1*06:02
540	DRB1*01:01	DRB1*03:01	DQB1*05:01	DQB1*02:01
541	DRB1*11:04	DRB1*15:01	DQB1*03:01	DQB1*06:02
542	DRB1*01:02	DRB1*13:01	DQB1*05:01	DQB1*06:03
543	DRB1*07:01	DRB1*14:01	DQB1*02:01	DQB1*05:03
544	DRB1*08:01	DRB1*11:02	DQB1*04:02	DQB1*03:01
545	DRB1*07:01	DRB1*13:05	DQB1*02:01	DQB1*03:01
546	DRB1*13:02	DRB1*15:03	DQB1*05:01	DQB1*06:02
547	DRB1*04:01	DRB1*08:07	DQB1*03:01	DQB1*04:02
548	DRB1*08:03	DRB1*11:01	DQB1*03:01	DQB1*06:02
549	DRB1*01:02	DRB1*03:01	DQB1*05:01	DQB1*02:01
550	DRB1*01:02	DRB1*12:01	DQB1*05:01	DQB1*03:01
551	DRB1*01:02	DRB1*16:01	DQB1*05:01	DQB1*05:02
552	DRB1*13:01	DRB1*15:01	DQB1*05:01	DQB1*06:02
553	DRB1*03:02	DRB1*10:01	DQB1*04:02	DQB1*05:01
554	DRB1*10:01	DRB1*12:01	DQB1*05:01	DQB1*05:01
555	DRB1*11:01	DRB1*13:02	DQB1*03:01	NA
556	DRB1*16:01	DRB1*07:01	DQB1*05:02	DQB1*02:01
557	DRB1*08:02	DRB1*13:02	DQB1*04:02	DQB1*05:01
558	DRB1*03:01	DRB1*15:01	DQB1*02:01	DQB1*06:02
559	DRB1*07:01	DRB1*08:04	DQB1*02:01	DQB1*04:02
560	DRB1*01:01	DRB1*15:01	DQB1*05:01	DQB1*06:02
561	DRB1*08:01	DRB1*13:03	DQB1*04:02	DQB1*03:01

562	DRB1*15:01	DRB1*15:02	DQB1*06:02	NA
563	DRB1*15:01	DRB1*07:01	DQB1*06:02	DQB1*02:01
564	DRB1*13:01	DRB1*13:02	DQB1*30:32	NA
565	DRB1*04:01	DRB1*11:01	DQB1*03:02	DQB1*03:01
566	DRB1*01:02	DRB1*11:02	DQB1*05:01	DQB1*03:01
567	DRB1*01:01	DRB1*12:01	DQB1*05:01	DQB1*05:01
568	DRB1*07:01	DRB1*13:02	DQB1*02:01	DQB1*06:04
569	DRB1*11:02	DRB1*11:04	DQB1*03:01	DQB1*06:03
570	DRB1*01:01	DRB1*01:01	DQB1*05:01	DQB1*05:01
571	DRB1*04:02	NA	DQB1*03:02	DQB1*06:05
572	DRB1*08:03	DRB1*11:02	DQB1*03:01	DQB1*03:01
573	DRB1*03:02	DRB1*13:01	DQB1*04:02	DQB1*06:03
574	DRB1*04:05	DRB1*11:02	DQB1*03:02	DQB1*03:01
575	DRB1*03:02	DRB1*07:01	DQB1*04:02	DQB1*02:01
576	DRB1*07:01	DRB1*07:01	DQB1*02:01	DQB1*02:01
577	DRB1*15:03	DRB1*11:02	DQB1*06:02	DQB1*03:01
578	DRB1*13:01	DRB1*14:01	DQB1*06:03	DQB1*05:01
579	DRB1*01:01	DRB1*07:01	DQB1*05:01	DQB1*02:01
580	DRB1*04:01	DRB1*15:01	DQB1*03:02	DQB1*06:02
581	DRB1*07:01	DRB1*11:01	DQB1*02:01	DQB1*06:02
582	DRB1*03:01	DRB1*13:01	DQB1*02:01	NA
583	DRB1*03:01	DRB1*13:01	DQB1*02:01	DQB1*06:03
584	DRB1*11:02	DRB1*13:02	DQB1*03:01	NA
585	DRB1*07:01	DRB1*13:01	DQB1*02:01	DQB1*06:03
586	DRB1*01:01	DRB1*04:02	DQB1*05:01	DQB1*03:02
587	DRB1*04:01	NA	DQB1*03:01	DQB1*05:03
588	DRB1*04:05	DRB1*07:01	DQB1*03:02	DQB1*02:01
589	DRB1*03:01	DRB1*03:01	DQB1*02:01	DQB1*02:01
590	DRB1*01:02	DRB1*03:01	DQB1*05:01	DQB1*02:01
591	DRB1*08:01	DRB1*11:03	DQB1*04:02	DQB1*03:01
592	DRB1*11:01	DRB1*15:01	DQB1*03:01	DQB1*06:02
593	DRB1*01:02	DRB1*13:01	DQB1*05:01	DQB1*06:03
594	DRB1*11:04	DRB1*13:02	DQB1*03:01	DQB1*06:01
595	DRB1*08:02	DRB1*08:04	DQB1*04:02	DQB1*04:02
596	DRB1*11:02	DRB1*07:01	DQB1*03:01	DQB1*02:01
597	DRB1*03:01	DRB1*16:01	DQB1*02:01	DQB1*05:02
598	DRB1*04:04	DRB1*11:02	DQB1*03:02	DQB1*03:01
599	DRB1*04:11	DRB1*13:01	DQB1*03:02	DQB1*06:03
600	DRB1*08:04	DRB1*08:04	DQB1*03:01	DQB1*04:02
601	DRB1*03:01	DRB1*14:01	DQB1*02:01	DQB1*05:03
602	DRB1*04:01	DRB1*11:01	NA	DQB1*03:01
603	DRB1*11:01	DRB1*13:01	DQB1*03:01	DQB1*06:03
604	DRB1*01:02	DRB1*08:01	DQB1*05:01	DQB1*04:02
605	DRB1*08:01	DRB1*10:01	DQB1*05:01	DQB1*03:01
606	DRB1*04:03	DRB1*14:01	DQB1*03:02	DQB1*05:03
607	DRB1*04:01	DRB1*03:01	DQB1*03:01	DQB1*02:01
608	DRB1*12:01	DRB1*13:02	DQB1*03:01	DQB1*06:04

609	DRB1*01:01	DRB1*15:01	DQB1*05:01	DQB1*06:02
610	DRB1*01:01	DRB1*04:02	DQB1*05:01	DQB1*03:02
611	DRB1*11:01	DRB1*13:02	DQB1*06:02	DQB1*06:04
612	DRB1*03:01	DRB1*13:02	DQB1*02:01	DQB1*06:04
613	DRB1*01:02	DRB1*15:03	DQB1*05:01	DQB1*06:02
614	DRB1*01:01	DRB1*15:03	DQB1*05:01	DQB1*06:02
615	DRB1*14:01	DRB1*10:01	DQB1*05:03	DQB1*05:01
616	DRB1*11:04	DRB1*15:03	DQB1*03:01	DQB1*06:02
617	DRB1*07:01	DRB1*07:01	DQB1*02:01	DQB1*02:01
618	DRB1*03:01	DRB1*15:01	DQB1*02:01	DQB1*06:02
619	DRB1*08:02	DRB1*09:01	DQB1*04:02	DQB1*30:32
620	DRB1*07:01	DRB1*13:01	DQB1*02:01	DQB1*06:04
621	DRB1*03:01	DRB1*09:01	DQB1*02:01	DQB1*30:32
622	DRB1*03:01	DRB1*07:01	DQB1*02:01	DQB1*02:01
623	DRB1*07:01	DRB1*11:03	DQB1*02:01	DQB1*03:01
624	DRB1*01:02	DRB1*04:07	DQB1*05:01	DQB1*03:02
625	DRB1*01:01	DRB1*08:01	DQB1*05:01	DQB1*04:02
626	DRB1*04:07	DRB1*04:11	DQB1*03:01	DQB1*03:02
627	DRB1*03:01	DRB1*14:02	DQB1*02:01	DQB1*03:01
628	DRB1*03:01	DRB1*15:01	DQB1*02:01	DQB1*06:02
629	DRB1*03:01	DRB1*13:03	DQB1*02:01	DQB1*03:01
630	DRB1*03:02	DRB1*04:11	DQB1*04:02	DQB1*03:02
631	DRB1*03:01	DRB1*13:01	DQB1*02:01	DQB1*06:03
632	DRB1*03:01	DRB1*03:01	DQB1*02:01	DQB1*02:01
633	DRB1*04:04	DRB1*03:01	DQB1*03:02	DQB1*02:01
634	DRB1*03:01	DRB1*11:02	DQB1*02:01	DQB1*03:01
635	DRB1*15:03	DRB1*11:01	DQB1*06:02	DQB1*06:02
636	DRB1*07:01	DRB1*08:04	DQB1*02:01	DQB1*03:01
637	DRB1*07:01	DRB1*13:03	DQB1*02:01	DQB1*03:01
638	DRB1*11:01	DRB1*13:02	DQB1*03:01	DQB1*06:04
639	DRB1*04:04	DRB1*07:01	DQB1*03:02	DQB1*02:01
640	DRB1*03:01	DRB1*11:01	DQB1*02:01	DQB1*03:01
641	DRB1*15:03	DRB1*03:02	DQB1*06:02	DQB1*04:02
642	DRB1*15:03	DRB1*16:02	DQB1*06:02	DQB1*05:02
643	DRB1*10:01	DRB1*01:01	DQB1*05:01	DQB1*05:01
644	DRB1*03:02	DRB1*08:04	DQB1*04:02	DQB1*04:02
645	DRB1*15:03	DRB1*03:01	DQB1*06:02	DQB1*02:01
646	DRB1*04:04	DRB1*16:02	DQB1*03:02	DQB1*05:02
647	DRB1*14:01	DRB1*13:04	DQB1*05:03	DQB1*03:01
648	DRB1*03:01	DRB1*13:02	DQB1*02:01	DQB1*06:05
649	DRB1*01:02	NA	DQB1*05:01	DQB1*04:02
650	DRB1*01:01	DRB1*08:04	DQB1*05:01	DQB1*03:01
651	DRB1*13:01	DRB1*13:01	DQB1*06:03	DQB1*06:03
652	DRB1*03:01	DRB1*13:02	DQB1*02:01	DQB1*06:05
653	DRB1*07:01	DRB1*07:01	DQB1*02:01	DQB1*02:01
654	DRB1*03:02	DRB1*13:01	DQB1*04:02	DQB1*06:03
655	DRB1*04:07	DRB1*15:01	DQB1*03:01	DQB1*06:02

656	DRB1*11:01	DRB1*15:01	DQB1*03:01	DQB1*06:02
657	DRB1*03:02	DRB1*07:01	DQB1*04:02	DQB1*30:32
658	DRB1*01:01	DRB1*11:01	DQB1*05:01	DQB1*06:02
659	NA	DRB1*01:01	DQB1*05:01	DQB1*05:01
660	DRB1*04:05	DRB1*15:01	DQB1*03:02	DQB1*05:02
661	DRB1*01:02	DRB1*04:02	DQB1*05:01	DQB1*03:02
662	DRB1*13:01	DRB1*13:03	DQB1*06:03	DQB1*02:01
663	DRB1*01:02	DRB1*03:01	DQB1*05:01	DQB1*02:01
664	DRB1*04:04	DRB1*15:03	DQB1*03:02	DQB1*06:02
665	DRB1*03:02	DRB1*15:03	DQB1*04:02	DQB1*06:02
666	DRB1*03:02	DRB1*08:04	DQB1*04:02	DQB1*03:01
667	DRB1*01:02	DRB1*03:01	DQB1*05:01	DQB1*02:01
668	DRB1*07:01	DRB1*15:01	DQB1*30:32	DQB1*06:02
669	DRB1*03:01	DRB1*08:01	DQB1*02:01	DQB1*04:02
670	DRB1*01:02	DRB1*03:02	DQB1*05:01	DQB1*04:02
671	DRB1*03:02	DRB1*15:01	DQB1*04:02	DQB1*06:02
672	DRB1*04:11	DRB1*07:01	DQB1*03:02	DQB1*02:01
673	DRB1*08:01	DRB1*14:02	DQB1*04:02	DQB1*03:01
674	DRB1*15:03	DRB1*14:01	DQB1*06:02	DQB1*05:01
675	DRB1*13:01	DRB1*15:03	DQB1*06:03	DQB1*06:02
676	DRB1*14:01	DRB1*14:02	NA	DQB1*03:01
677	DRB1*11:01	DRB1*07:01	DQB1*06:02	DQB1*02:01
678	DRB1*04:11	DRB1*03:02	NA	DQB1*04:02
679	DRB1*11:01	DRB1*11:01	DQB1*03:01	DQB1*03:01
680	DRB1*09:01	DRB1*07:01	DQB1*02:01	DQB1*02:01
681	DRB1*01:02	DRB1*13:02	DQB1*05:01	DQB1*06:05
682	DRB1*13:01	DRB1*16:01	DQB1*06:03	DQB1*05:02
683	DRB1*04:03	DRB1*07:01	DQB1*03:02	DQB1*02:01
684	DRB1*13:01	DRB1*10:01	DQB1*06:03	DQB1*05:01
685	DRB1*01:01	DRB1*16:01	DQB1*05:01	DQB1*05:02
686	DRB1*11:01	DRB1*13:01	DQB1*05:02	DQB1*06:03
687	DRB1*03:02	DRB1*13:01	DQB1*04:02	DQB1*06:03
688	DRB1*04:04	DRB1*08:07	DQB1*03:02	DQB1*04:02
689	DRB1*13:03	DRB1*07:01	DQB1*03:01	DQB1*02:01
690	DRB1*08:02	DRB1*16:02	DQB1*04:02	DQB1*03:01
691	DRB1*03:03	DRB1*13:02	DQB1*04:02	DQB1*06:04
692	DRB1*04:04	DRB1*13:01	DQB1*03:02	DQB1*06:03
693	DRB1*13:01	DRB1*13:01	DQB1*06:03	DQB1*06:04
694	DRB1*04:11	DRB1*13:03	DQB1*03:02	DQB1*02:01
695	DRB1*04:07	DRB1*04:07	DQB1*03:01	DQB1*03:01
696	DRB1*14:02	DRB1*07:01	DQB1*03:01	DQB1*02:01
697	DRB1*07:01	DRB1*16:02	DQB1*02:01	DQB1*03:01
698	DRB1*01:02	NA	DQB1*05:01	DQB1*03:01
699	DRB1*15:01	DRB1*13:01	DQB1*06:02	DQB1*05:01
700	DRB1*04:05	DRB1*09:01	DQB1*03:02	DQB1*02:01
701	DRB1*08:02	DRB1*12:01	DQB1*04:02	DQB1*05:01
702	DRB1*11:01	DRB1*15:03	DQB1*06:02	DQB1*06:02

703	DRB1*13:01	NA	DQB1*03:01	DQB1*02:01
704	DRB1*01:02	DRB1*04:01	DQB1*05:01	DQB1*03:02
705	DRB1*07:01	DRB1*13:01	DQB1*02:01	DQB1*06:03
706	DRB1*13:02	DRB1*15:01	DQB1*06:04	DQB1*06:02
707	DRB1*08:04	DRB1*09:01	DQB1*03:01	DQB1*30:32
708	DRB1*01:02	DRB1*13:01	DQB1*05:01	DQB1*06:03
709	DRB1*03:01	DRB1*11:01	DQB1*02:01	DQB1*05:02
710	DRB1*11:01	NA	DQB1*06:02	DQB1*30:32
711	DRB1*09:01	DRB1*07:01	NA	DQB1*02:01
712	DRB1*04:05	DRB1*07:01	DQB1*03:02	DQB1*02:01
713	DRB1*03:02	DRB1*16:02	DQB1*04:02	DQB1*05:02
714	DRB1*15:03	DRB1*03:01	DQB1*06:02	DQB1*02:01
715	DRB1*03:01	DRB1*13:02	DQB1*02:01	DQB1*06:04
716	DRB1*12:02	DRB1*07:01	DQB1*05:01	DQB1*02:01
717	DRB1*03:01	DRB1*11:02	DQB1*02:01	DQB1*03:01
718	DRB1*04:03	DRB1*14:01	DQB1*03:04	DQB1*05:03
719	DRB1*03:01	DRB1*11:03	DQB1*02:01	DQB1*03:01
720	DRB1*01:02	DRB1*08:01	DQB1*05:01	DQB1*04:02
721	DRB1*04:02	DRB1*13:02	DQB1*30:32	DQB1*06:04
722	DRB1*03:01	DRB1*09:01	DQB1*02:01	DQB1*02:01
723	DRB1*11:01	DRB1*13:01	DQB1*06:02	DQB1*06:02
724	DRB1*07:01	DRB1*11:01	DQB1*02:01	DQB1*03:01
725	DRB1*11:01	NA	DQB1*03:01	NA
726	DRB1*07:01	DRB1*09:01	DQB1*02:01	DQB1*30:32
727	DRB1*04:07	NA	DQB1*03:01	DQB1*05:01
728	DRB1*01:01	DRB1*08:02	DQB1*05:01	DQB1*04:02
729	DRB1*03:01	DRB1*11:01	DQB1*02:01	DQB1*03:01
730	DRB1*15:01	DRB1*15:01	DQB1*06:01	DQB1*06:02
731	DRB1*03:01	DRB1*11:02	DQB1*02:01	DQB1*03:01
732	DRB1*01:01	DRB1*03:02	DQB1*05:01	DQB1*04:02
733	DRB1*03:01	DRB1*11:04	DQB1*02:01	DQB1*03:01
734	DRB1*03:01	DRB1*07:01	DQB1*02:01	DQB1*02:01
735	DRB1*07:01	DRB1*15:03	DQB1*02:01	DQB1*06:02
736	DRB1*01:02	DRB1*09:01	DQB1*05:01	DQB1*02:01
737	DRB1*03:01	DRB1*13:01	DQB1*02:01	DQB1*06:03
738	DRB1*03:01	DRB1*07:01	DQB1*02:01	DQB1*02:01
739	NA	NA	DQB1*30:32	DQB1*05:01
740	DRB1*01:01	DRB1*08:04	DQB1*05:01	DQB1*03:01
741	DRB1*15:03	DRB1*10:01	DQB1*06:02	DQB1*05:01
742	DRB1*11:01	DRB1*15:01	DQB1*03:01	DQB1*06:02
743	DRB1*04:01	DRB1*16:02	DQB1*03:01	DQB1*03:01
744	DRB1*07:01	DRB1*11:04	DQB1*02:01	DQB1*05:02
745	DRB1*15:01	DRB1*16:02	DQB1*06:02	DQB1*03:01
746	DRB1*03:01	DRB1*11:02	DQB1*02:01	DQB1*03:01
747	DRB1*07:01	DRB1*15:01	DQB1*02:01	DQB1*06:02
748	DRB1*15:01	DRB1*11:02	DQB1*06:02	DQB1*03:01
749	DRB1*13:02	DRB1*15:03	DQB1*06:04	DQB1*06:02

750	DRB1*03:02	DRB1*04:11	DQB1*04:02	DQB1*03:02
751	DRB1*01:02	NA	DQB1*05:01	DQB1*06:02
752	DRB1*14:02	DRB1*07:01	DQB1*03:01	DQB1*02:01
753	DRB1*01:02	DRB1*01:02	DQB1*05:01	DQB1*05:01
754	DRB1*14:01	DRB1*13:02	DQB1*05:03	DQB1*06:04
755	DRB1*01:02	DRB1*13:03	DQB1*05:01	DQB1*03:01
756	DRB1*03:02	DRB1*13:02	DQB1*04:02	DQB1*05:01
757	DRB1*11:04	DRB1*07:01	DQB1*03:01	DQB1*02:01
758	DRB1*01:01	DRB1*07:01	DQB1*05:01	NA
759	DRB1*01:02	DRB1*07:01	DQB1*05:01	DQB1*02:01
760	DRB1*11:01	DRB1*11:01	DQB1*06:02	DQB1*03:01
761	DRB1*07:01	DRB1*15:03	DQB1*02:01	DQB1*06:02
762	DRB1*08:01	DRB1*13:01	DQB1*04:02	DQB1*06:03
763	DRB1*11:02	DRB1*15:03	DQB1*03:01	DQB1*06:02
764	DRB1*15:01	DRB1*04:01	NA	NA
765	DRB1*04:05	DRB1*08:04	DQB1*03:02	DQB1*03:01
766	DRB1*04:04	DRB1*03:01	DQB1*03:02	DQB1*02:01
767	DRB1*16:01	DRB1*03:01	DQB1*05:02	DQB1*02:01
768	DRB1*07:01	DRB1*11:03	DQB1*02:01	DQB1*03:01
769	DRB1*01:01	DRB1*04:05	NA	NA
770	NA	NA	DQB1*04:02	NA
771	DRB1*01:03	DRB1*13:01	DQB1*05:01	DQB1*06:03
772	DRB1*03:02	DRB1*11:01	DQB1*04:02	DQB1*06:02
773	DRB1*03:01	DRB1*03:02	DQB1*02:01	DQB1*04:02
774	DRB1*01:02	DRB1*11:01	DQB1*05:01	DQB1*03:01
775	DRB1*04:03	DRB1*13:03	DQB1*03:02	DQB1*03:01
776	DRB1*15:03	DRB1*03:01	DQB1*06:02	DQB1*02:01
777	DRB1*04:04	NA	NA	DQB1*05:03
778	DRB1*04:02	DRB1*13:01	DQB1*03:02	DQB1*06:03
779	DRB1*01:02	DRB1*03:02	DQB1*05:01	DQB1*04:02
780	DRB1*15:01	DRB1*04:08	DQB1*05:01	NA
781	DRB1*07:01	DRB1*15:03	DQB1*02:01	DQB1*06:02
782	DRB1*11:01	DRB1*13:01	DQB1*03:01	DQB1*06:03
783	DRB1*01:03	DRB1*13:01	NA	DQB1*06:04
784	DRB1*08:07	DRB1*13:01	DQB1*04:02	DQB1*06:03
785	DRB1*15:01	DRB1*04:04	DQB1*06:02	DQB1*03:02
786	DRB1*03:01	DRB1*07:01	DQB1*02:01	DQB1*02:01
787	DRB1*07:01	DRB1*04:11	DQB1*02:01	DQB1*03:02
788	DRB1*13:01	DRB1*07:01	DQB1*05:01	DQB1*02:01
789	DRB1*08:02	DRB1*14:01	DQB1*04:02	DQB1*05:03
790	DRB1*01:01	DRB1*08:01	NA	DQB1*04:02
791	DRB1*01:01	DRB1*14:01	DQB1*05:01	DQB1*05:03
792	DRB1*01:03	DRB1*13:02	DQB1*05:01	NA
793	DRB1*04:02	DRB1*13:01	DQB1*03:02	DQB1*06:03
794	DRB1*01:02	DRB1*16:01	DQB1*05:01	DQB1*05:02
795	DRB1*01:01	DRB1*04:01	DQB1*05:01	NA
796	DRB1*03:01	DRB1*04:03	DQB1*02:01	DQB1*03:02

797	DRB1*01:01	DRB1*16:01	DQB1*05:01	NA
798	DRB1*01:02	DRB1*07:01	DQB1*05:01	DQB1*02:01
799	DRB1*15:03	DRB1*03:01	DQB1*06:02	NA
800	DRB1*11:01	DRB1*07:01	DQB1*06:02	DQB1*02:01
801	DRB1*08:01	NA	DQB1*04:02	DQB1*06:02
802	DRB1*01:01	DRB1*07:01	DQB1*05:01	DQB1*02:01
803	DRB1*13:01	DRB1*07:01	DQB1*03:01	DQB1*02:01
804	DRB1*09:01	NA	DQB1*02:01	DQB1*03:02
805	DRB1*04:02	DRB1*14:01	DQB1*03:02	DQB1*05:03
806	DRB1*03:01	NA	DQB1*02:01	NA
807	DRB1*03:01	DRB1*13:02	DQB1*02:01	NA
808	NA	DRB1*07:01	NA	NA
809	DRB1*15:03	DRB1*03:01	DQB1*06:02	DQB1*02:01
810	DRB1*04:03	DRB1*09:01	DQB1*03:02	DQB1*02:01
811	DRB1*03:01	DRB1*11:04	DQB1*02:01	DQB1*03:01
812	DRB1*15:01	DRB1*11:01	DQB1*06:02	DQB1*03:01
813	DRB1*04:01	DRB1*07:01	DQB1*03:02	DQB1*02:01
814	DRB1*15:03	DRB1*07:01	DQB1*06:02	DQB1*02:01
815	NA	NA	DQB1*30:32	DQB1*03:02
816	DRB1*03:01	DRB1*11:04	DQB1*02:01	DQB1*03:01
817	DRB1*07:01	DRB1*07:01	DQB1*02:01	DQB1*02:01
818	DRB1*04:01	DRB1*07:01	DQB1*03:01	DQB1*02:01
819	DRB1*04:01	DRB1*07:01	DQB1*03:01	DQB1*02:01
820	DRB1*03:01	DRB1*13:01	DQB1*02:01	DQB1*06:02
821	NA	NA	DQB1*04:02	DQB1*02:01
822	DRB1*04:05	DRB1*07:01	DQB1*03:02	DQB1*30:32
823	DRB1*04:11	NA	DQB1*03:02	NA
824	DRB1*04:05	NA	DQB1*30:32	NA
825	DRB1*03:01	DRB1*07:01	DQB1*02:01	DQB1*02:01
826	DRB1*15:01	DRB1*11:04	DQB1*06:02	DQB1*03:01
827	DRB1*04:01	DRB1*13:01	DQB1*03:02	DQB1*06:03
828	DRB1*16:02	DRB1*07:01	DQB1*05:02	DQB1*02:01
829	NA	NA	DQB1*03:02	DQB1*03:02
830	DRB1*04:02	DRB1*15:01	DQB1*03:02	DQB1*06:02
831	DRB1*14:02	NA	DQB1*03:01	NA
832	DRB1*01:02	DRB1*04:11	DQB1*05:01	DQB1*03:02
833	DRB1*13:01	NA	DQB1*06:02	DQB1*30:32
834	DRB1*11:01	DRB1*13:02	DQB1*06:02	DQB1*05:01
835	DRB1*01:02	DRB1*13:01	DQB1*05:01	DQB1*06:03
836	DRB1*01:03	DRB1*13:02	DQB1*05:01	DQB1*06:03
837	DRB1*14:01	DRB1*07:01	DQB1*05:03	DQB1*02:01
838	DRB1*04:11	NA	NA	DQB1*04:02
839	DRB1*03:01	DRB1*08:01	DQB1*02:01	DQB1*04:02
840	DRB1*01:01	NA	NA	NA
841	DRB1*15:01	DRB1*13:01	DQB1*06:02	DQB1*06:04
842	DRB1*08:01	NA	DQB1*04:02	NA
843	DRB1*15:03	DRB1*07:01	DQB1*06:02	DQB1*02:01

844	DRB1*04:02	DRB1*03:02	DQB1*03:02	DQB1*04:02
845	DRB1*07:01	DRB1*13:02	DQB1*02:01	DQB1*06:04
846	DRB1*03:01	DRB1*13:02	DQB1*02:01	DQB1*06:04
847	DRB1*03:02	DRB1*10:01	DQB1*04:02	DQB1*05:01
848	DRB1*03:01	DRB1*13:01	DQB1*02:01	DQB1*06:03
849	NA	DRB1*15:01	DQB1*05:02	DQB1*06:02
850	DRB1*11:01	NA	DQB1*06:02	DQB1*03:01
851	DRB1*13:01	DRB1*07:01	NA	DQB1*30:32
852	DRB1*13:01	DRB1*13:02	DQB1*06:03	DQB1*06:04
853	DRB1*01:01	DRB1*13:01	DQB1*05:01	DQB1*06:03
854	DRB1*07:01	DRB1*11:01	DQB1*02:01	DQB1*03:01
855	DRB1*11:04	NA	DQB1*03:01	DQB1*05:02
856	DRB1*03:01	DRB1*08:07	DQB1*02:01	DQB1*04:02
857	DRB1*08:04	DRB1*07:01	DQB1*04:02	DQB1*02:01
858	DRB1*07:01	DRB1*07:01	DQB1*02:01	DQB1*02:01
859	DRB1*03:01	DRB1*07:01	DQB1*02:01	DQB1*02:01
860	DRB1*01:01	DRB1*07:01	DQB1*05:01	DQB1*02:01
861	DRB1*04:01	DRB1*07:01	DQB1*03:01	DQB1*02:01
862	DRB1*13:02	DRB1*07:01	DQB1*06:04	DQB1*02:01
863	DRB1*04:02	DRB1*12:02	DQB1*03:02	NA
864	DRB1*16:01	DRB1*13:01	DQB1*05:02	DQB1*06:03
865	DRB1*13:01	DRB1*13:03	DQB1*05:01	DQB1*02:01
866	DRB1*03:01	DRB1*08:01	DQB1*02:01	NA
867	DRB1*03:01	DRB1*15:03	DQB1*02:01	DQB1*06:02
868	DRB1*03:01	DRB1*11:02	DQB1*02:01	DQB1*03:01
869	DRB1*11:01	DRB1*13:01	DQB1*03:01	DQB1*06:03
870	DRB1*01:02	DRB1*01:02	DQB1*05:01	DQB1*05:01
871	DRB1*08:01	DRB1*08:02	DQB1*06:02	DQB1*04:02
872	DRB1*10:01	DRB1*07:01	DQB1*05:01	DQB1*30:32
873	DRB1*11:02	DRB1*10:01	DQB1*03:01	DQB1*05:01
874	DRB1*03:01	DRB1*11:01	DQB1*02:01	DQB1*03:01
875	DRB1*01:01	DRB1*08:04	DQB1*05:01	DQB1*03:01
876	DRB1*13:01	DRB1*07:01	DQB1*06:03	DQB1*02:01
877	DRB1*15:02	DRB1*07:01	NA	DQB1*02:01
878	DRB1*15:01	DRB1*07:01	NA	DQB1*02:01
879	DRB1*09:01	NA	DQB1*30:32	DQB1*05:01
880	DRB1*03:01	DRB1*11:01	DQB1*02:01	DQB1*03:01
881	DRB1*01:01	DRB1*04:05	DQB1*05:01	DQB1*03:01
882	DRB1*04:11	DRB1*08:07	DQB1*03:02	DQB1*04:02
883	DRB1*15:03	DRB1*03:01	DQB1*06:02	DQB1*02:01
884	DRB1*03:01	DRB1*11:01	DQB1*02:01	DQB1*03:01
885	DRB1*11:02	DRB1*07:01	DQB1*03:01	DQB1*02:01
886	DRB1*11:02	DRB1*15:01	DQB1*03:01	DQB1*06:02
887	DRB1*16:02	DRB1*07:01	DQB1*05:02	DQB1*02:01
888	DRB1*13:01	DRB1*07:01	NA	DQB1*02:01
889	DRB1*11:04	DRB1*13:03	DQB1*03:01	DQB1*03:01
890	DRB1*01:02	DRB1*07:01	DQB1*05:01	DQB1*02:01

891	DRB1*11:01	NA	DQB1*03:01	DQB1*06:05
892	DRB1*15:01	DRB1*10:01	DQB1*06:02	DQB1*05:01
893	DRB1*15:01	DRB1*16:02	DQB1*06:02	DQB1*03:01
894	DRB1*15:01	DRB1*11:01	DQB1*06:02	DQB1*03:01
895	DRB1*13:01	DRB1*10:01	DQB1*06:04	DQB1*05:01
896	DRB1*04:07	DRB1*03:01	NA	DQB1*02:01
897	DRB1*04:09	DRB1*15:01	DQB1*03:01	DQB1*06:02
898	DRB1*09:01	DRB1*15:03	DQB1*30:32	DQB1*06:02
899	DRB1*15:03	NA	DQB1*06:02	NA
900	DRB1*08:04	DRB1*13:03	DQB1*04:02	NA
901	DRB1*15:03	DRB1*03:01	DQB1*06:02	DQB1*02:01
902	DRB1*16:01	DRB1*07:01	DQB1*05:02	DQB1*30:32
903	DRB1*03:01	DRB1*03:01	DQB1*02:01	DQB1*02:01
904	DRB1*13:01	DRB1*13:02	DQB1*06:03	NA
905	DRB1*04:02	DRB1*11:01	DQB1*03:02	DQB1*03:01
906	DRB1*01:02	NA	DQB1*05:01	DQB1*03:01
907	DRB1*03:01	NA	DQB1*02:01	DQB1*06:03
908	DRB1*03:01	NA	DQB1*02:01	NA
909	DRB1*01:03	DRB1*11:01	DQB1*05:01	DQB1*06:02
910	DRB1*01:02	DRB1*11:01	DQB1*05:01	DQB1*03:01
911	DRB1*15:03	DRB1*11:01	DQB1*06:02	DQB1*03:01
912	DRB1*15:01	DRB1*07:01	DQB1*06:02	DQB1*02:01
913	DRB1*01:01	DRB1*13:02	DQB1*05:01	DQB1*06:04
914	DRB1*04:11	DRB1*13:02	DQB1*03:02	NA
915	DRB1*01:03	DRB1*15:01	NA	NA
916	DRB1*16:02	DRB1*04:01	DQB1*03:01	DQB1*03:01
917	DRB1*01:02	DRB1*08:04	DQB1*05:01	DQB1*03:01
918	DRB1*01:01	DRB1*03:01	DQB1*05:01	DQB1*02:01
919	DRB1*13:02	DRB1*09:01	DQB1*06:04	DQB1*02:01
920	DRB1*15:03	DRB1*10:01	DQB1*06:02	DQB1*05:01
921	DRB1*04:11	NA	DQB1*03:02	NA
922	DRB1*15:01	DRB1*04:11	DQB1*06:02	DQB1*03:02
923	DRB1*03:01	DRB1*13:05	DQB1*02:01	DQB1*03:01
924	DRB1*01:01	DRB1*07:01	NA	DQB1*02:01
925	NA	NA	DQB1*04:02	DQB1*06:03
926	DRB1*11:02	NA	DQB1*03:01	DQB1*02:01
927	DRB1*03:02	DRB1*13:02	DQB1*04:02	DQB1*05:01
928	DRB1*04:11	DRB1*03:01	DQB1*03:02	DQB1*02:01
929	DRB1*14:01	DRB1*07:01	DQB1*05:03	DQB1*02:01
930	DRB1*16:02	DRB1*13:02	NA	DQB1*05:01
931	DRB1*03:01	DRB1*10:01	DQB1*02:01	DQB1*05:01
932	DRB1*04:06	DRB1*11:01	DQB1*30:32	DQB1*04:02
933	DRB1*04:04	DRB1*10:01	DQB1*03:02	DQB1*05:01
934	DRB1*01:01	DRB1*07:01	DQB1*05:01	DQB1*02:01
935	DRB1*12:02	NA	DQB1*03:01	DQB1*05:01
936	DRB1*04:03	DRB1*09:01	DQB1*03:02	DQB1*02:01
937	DRB1*12:02	DRB1*07:01	DQB1*03:01	DQB1*02:01

938	DRB1*03:01	DRB1*08:04	DQB1*02:01	DQB1*03:01
939	DRB1*07:01	DRB1*07:01	DQB1*02:01	DQB1*02:01
940	DRB1*09:01	DRB1*07:01	DQB1*02:01	DQB1*02:01
941	DRB1*07:01	DRB1*07:01	DQB1*30:32	DQB1*02:01
942	DRB1*08:07	DRB1*11:01	DQB1*04:02	DQB1*03:01
943	DRB1*01:02	DRB1*03:01	DQB1*05:01	DQB1*02:01
944	DRB1*03:01	DRB1*13:02	DQB1*02:01	NA
945	DRB1*07:01	DRB1*12:01	DQB1*02:01	DQB1*03:01
946	DRB1*01:01	DRB1*11:01	DQB1*05:01	NA
947	DRB1*08:01	NA	DQB1*04:02	NA
948	DRB1*12:02	DRB1*14:01	DQB1*05:01	DQB1*05:03
949	DRB1*16:01	DRB1*07:01	DQB1*05:02	NA
950	DRB1*15:03	DRB1*08:06	DQB1*06:02	DQB1*03:01
951	DRB1*13:01	NA	NA	DQB1*05:02
952	DRB1*11:02	DRB1*13:03	NA	DQB1*02:01
953	DRB1*15:03	DRB1*03:02	DQB1*06:02	DQB1*04:02
954	DRB1*03:01	DRB1*11:02	DQB1*02:01	DQB1*03:01
955	DRB1*04:02	NA	DQB1*30:32	NA
956	DRB1*07:01	DRB1*07:01	DQB1*30:32	DQB1*30:32
957	DRB1*04:01	DRB1*04:04	DQB1*03:02	DQB1*03:02
958	DRB1*01:02	DRB1*15:03	DQB1*05:01	DQB1*06:02
959	DRB1*08:04	DRB1*12:01	DQB1*04:02	DQB1*05:01
960	DRB1*11:01	DRB1*11:01	DQB1*03:01	DQB1*06:02
961	DRB1*01:03	DRB1*12:02	DQB1*05:01	NA
962	DRB1*08:01	DRB1*07:01	DQB1*04:02	DQB1*02:01
963	DRB1*04:05	DRB1*03:01	NA	DQB1*02:01
964	DRB1*01:01	DRB1*08:04	DQB1*05:01	NA
965	DRB1*09:01	DRB1*07:01	DQB1*02:01	DQB1*02:01
966	DRB1*01:02	DRB1*10:01	DQB1*05:01	DQB1*05:01
967	DRB1*01:01	DRB1*13:02	DQB1*05:01	DQB1*05:01
968	DRB1*15:01	NA	DQB1*06:02	NA
969	DRB1*12:02	DRB1*14:01	DQB1*30:32	DQB1*05:03
970	DRB1*01:01	DRB1*03:01	DQB1*05:01	DQB1*02:01
971	DRB1*15:01	DRB1*10:01	DQB1*06:02	DQB1*05:01
972	DRB1*16:02	DRB1*11:04	DQB1*05:02	DQB1*03:01
973	DRB1*01:03	DRB1*11:01	DQB1*05:01	DQB1*06:02
974	DRB1*11:01	DRB1*07:01	DQB1*06:02	DQB1*02:01
975	DRB1*04:11	DRB1*13:01	NA	DQB1*06:03
976	DRB1*01:02	DRB1*15:01	DQB1*05:01	DQB1*06:02
977	DRB1*16:01	DRB1*11:01	DQB1*05:02	DQB1*03:01
978	DRB1*04:05	DRB1*12:02	NA	NA
979	DRB1*15:03	DRB1*13:01	DQB1*06:02	DQB1*05:02
980	DRB1*15:01	NA	DQB1*06:02	NA
981	DRB1*04:03	DRB1*12:02	NA	NA
982	DRB1*03:01	NA	DQB1*02:01	DQB1*06:04
983	DRB1*03:01	DRB1*11:01	DQB1*02:01	DQB1*03:01
984	DRB1*01:03	DRB1*15:02	DQB1*05:01	NA

985	DRB1*04:05	DRB1*11:02	DQB1*30:32	NA
986	DRB1*01:03	DRB1*04:07	DQB1*05:01	DQB1*03:02
987	DRB1*01:03	DRB1*13:01	DQB1*05:03	DQB1*06:03
988	DRB1*15:01	DRB1*11:02	DQB1*06:02	DQB1*03:01
989	DRB1*11:01	DRB1*11:01	DQB1*03:01	NA
990	DRB1*01:02	NA	DQB1*05:01	NA
991	DRB1*15:01	NA	DQB1*06:02	DQB1*05:02
992	DRB1*03:01	DRB1*09:01	DQB1*02:01	DQB1*02:01
993	DRB1*04:04	DRB1*13:02	DQB1*06:04	DQB1*06:04
994	DRB1*01:02	DRB1*11:01	DQB1*05:01	NA
995	DRB1*11:01	DRB1*11:04	DQB1*03:01	DQB1*03:01
996	DRB1*04:04	DRB1*07:01	DQB1*03:02	DQB1*02:01
997	DRB1*15:03	DRB1*15:03	DQB1*06:02	DQB1*06:02
998	DRB1*11:01	DRB1*11:02	DQB1*03:01	DQB1*03:01
999	DRB1*15:01	DRB1*11:01	DQB1*06:02	NA
1000	DRB1*16:01	DRB1*07:01	DQB1*05:02	DQB1*02:01
1001	DRB1*11:01	DRB1*07:01	DQB1*06:02	DQB1*02:01
1002	DRB1*04:04	DRB1*13:01	DQB1*03:02	DQB1*06:03
1003	DRB1*15:01	DRB1*04:01	DQB1*06:02	DQB1*03:01
1004	DRB1*11:04	NA	DQB1*03:01	DQB1*05:03
1005	DRB1*01:01	DRB1*04:03	DQB1*05:01	NA
1006	DRB1*15:01	DRB1*07:01	DQB1*06:02	DQB1*02:01
1007	DRB1*04:11	DRB1*08:04	DQB1*03:02	DQB1*03:01
1008	DRB1*11:01	DRB1*11:02	NA	DQB1*03:01
1009	DRB1*01:03	DRB1*04:07	DQB1*05:01	DQB1*03:02
1010	DRB1*04:04	DRB1*07:01	DQB1*03:02	DQB1*30:32
1011	DRB1*11:04	DRB1*07:01	DQB1*03:01	DQB1*02:01
1012	DRB1*08:01	DRB1*07:01	DQB1*04:02	DQB1*02:01
1013	DRB1*15:03	DRB1*13:01	DQB1*06:02	DQB1*06:03
1014	DRB1*03:02	DRB1*07:01	DQB1*04:02	DQB1*02:01
1015	DRB1*15:03	DRB1*03:02	DQB1*06:02	NA
1016	DRB1*01:02	DRB1*13:01	DQB1*05:01	DQB1*06:03
1017	DRB1*11:01	NA	DQB1*03:01	DQB1*05:01
1018	DRB1*13:01	NA	DQB1*06:03	DQB1*06:03
1019	DRB1*07:01	DRB1*07:01	DQB1*02:01	DQB1*02:01
1020	DRB1*11:04	NA	DQB1*03:01	DQB1*03:01
1021	DRB1*01:01	NA	DQB1*05:01	DQB1*05:02
1022	DRB1*16:01	DRB1*03:02	DQB1*05:02	DQB1*04:02
1023	DRB1*15:01	DRB1*16:01	DQB1*06:02	DQB1*05:02
1024	DRB1*11:01	DRB1*09:01	DQB1*06:02	DQB1*02:01
1025	DRB1*04:02	DRB1*03:01	NA	DQB1*02:01
1026	DRB1*01:03	DRB1*04:11	DQB1*05:01	DQB1*03:02
1027	DRB1*03:01	DRB1*10:01	DQB1*02:01	NA
1028	DRB1*04:05	DRB1*07:01	DQB1*02:01	DQB1*02:01
1029	DRB1*03:02	DRB1*09:01	DQB1*04:02	DQB1*02:01

NA: not available
